# Neurexin and neuroligins jointly regulate synaptic degeneration at the *Drosophila* neuromuscular junction based on TEM studies

**DOI:** 10.3389/fncel.2023.1257347

**Published:** 2023-11-01

**Authors:** Gan Guangming, Chen Mei, Yu Qinfeng, Gao Xiang, Zhang Chenchen, Sheng Qingyuan, Xie Wei, Geng Junhua

**Affiliations:** ^1^School of Medicine, Southeast University, Nanjing, Jiangsu, China; ^2^School of Life Science and Technology, The Key Laboratory of Developmental Genes and Human Disease, Southeast University, Nanjing, Jiangsu, China; ^3^The Collaborative Innovation Center for Brain Science, Southeast University, Nanjing, Jiangsu, China; ^4^Shenzhen Research Institute of Southeast University, Shenzhen, Guangdong, China

**Keywords:** *Drosophila*, neuromuscular junction, synaptic degeneration, neurexin, neuroligins, transmission electron microscopy

## Abstract

The *Drosophila* larval neuromuscular junction (NMJ) is a well-known model system and is often used to study synapse development. Here, we show synaptic degeneration at NMJ boutons, primarily based on transmission electron microscopy (TEM) studies. When degeneration starts, the subsynaptic reticulum (SSR) swells, retracts and folds inward, and the residual SSR then degenerates into a disordered, thin or linear membrane. The axon terminal begins to degenerate from the central region, and the T-bar detaches from the presynaptic membrane with clustered synaptic vesicles to accelerate large-scale degeneration. There are two degeneration modes for clear synaptic vesicles. In the first mode, synaptic vesicles without actin filaments degenerate on the membrane with ultrafine spots and collapse and disperse to form an irregular profile with dark ultrafine particles. In the second mode, clear synaptic vesicles with actin filaments degenerate into dense synaptic vesicles, form irregular dark clumps without a membrane, and collapse and disperse to form an irregular profile with dark ultrafine particles. Last, all residual membranes in NMJ boutons degenerate into a linear shape, and all the residual elements in axon terminals degenerate and eventually form a cluster of dark ultrafine particles. Swelling and retraction of the SSR occurs prior to degradation of the axon terminal, which degenerates faster and with more intensity than the SSR. NMJ bouton degeneration occurs under normal physiological conditions but is accelerated in *Drosophila neurexin* (*dnrx*) *dnrx^273^*, *Drosophila neuroligin* (*dnlg*) *dnlg1* and *dnlg4* mutants and *dnrx^83^*;*dnlg3* and *dnlg2*;*dnlg3* double mutants, which suggests that both neurexin and neuroligins play a vital role in preventing synaptic degeneration.

## Background

Efficient signal communication is carried out by means of a massive number of delicate synapses among neurons. During nervous system development, neurons tend to produce redundant synaptic connections that will be pruned (Watts et al., 2004; Awasaki et al., 2006) or degenerated (Siskova et al., 2010; Cao et al., 2013; Lu et al., 2017; Perry et al., 2017) and then eliminated (Mikuni et al., 2013; Wilkerson et al., 2014) by astrocytes (Chung et al., 2013; Tasdemir-Yilmaz and Freeman, 2014; Yang et al., 2016), microglia (Siskova et al., 2009; Miyamoto et al., 2016), and Schwann cells (Gillingwater et al., 2002; Smith et al., 2013; Lee et al., 2016; Jung et al., 2019). Therefore, the stability of synaptic boutons is a dynamic balance among growth (Miyamoto et al., 2016), pruning, degeneration, and elimination processes. Synapse degeneration is a complicated process that includes retraction (Watts et al., 2003; Pielage et al., 2011) and degradation (Smith et al., 2013; Tan et al., 2017) of presynaptic and postsynaptic components, such as synaptic vesicles (Gillingwater et al., 2002; Siskova et al., 2009; Pielage et al., 2011; Qi et al., 2017; Fan et al., 2018; Pareek et al., 2018), microtubules (Awasaki et al., 2006), and postsynaptic density (Caleo et al., 2012). In most of synaptic degeneration, the degenerated ultrastructure shows the characteristics of dark electron density, which is very similar to the aging and death of organelles.

Disordered elimination after synaptic degeneration can lead to autism (Tang et al., 2014) and other neurological diseases. Loss of neurons in the brain and spinal cord leads to neurodegenerative diseases, such as Alzheimer’s disease, Parkinson’s disease, and amyotrophic lateral sclerosis. In the peripheral nervous system, degeneration of neuromuscular junctions (NMJs) occurs prior to cell soma degeneration (Frey et al., 2000; Fischer et al., 2004). Transmission electron microscopy (TEM) is a powerful tool for studying synaptic degeneration due to its ultrahigh resolution. In degenerating terminals, the synaptic vesicles will be reduced and collapse, with shrinking of the synaptic terminal (Siskova et al., 2009; Pielage et al., 2011). However, current studies of synaptic bouton degeneration primarily focus on the degradation of synapses and mitochondria, cytoskeletal disorders, and reductions in synaptic vesicles, while few studies have reported on the collapse and degradation of synaptic vesicles. The morphological collapse and degradation of synaptic vesicles is rarely reported in the literature and can technically be investigated using only electron microscopy.

The *Drosophila* larval NMJ is a well-known model system for studying synaptic development, signal transmission and neurological disease. Synapse retraction often precedes synapse degeneration in NMJ boutons in *Drosophila*. Synaptic debris and synaptic footprints are remnants of presynaptic boutons that retract from normal NMJ boutons in *Drosophila*. Synaptic debris are small in size and lack synapsin as a marker of clear synaptic vesicles and the postsynaptic Dlg protein as a marker of the NMJ bouton subsynaptic reticulum (SSR) but have obvious Hrp signals (Fuentes-Medel et al., 2009; Sutcliffe et al., 2013). Synaptic footprints with SSR membranes and retracted axon terminals have postsynaptic Dlg proteins (Eaton et al., 2002; Eaton and Davis, 2005) but are almost completely lacking synapsin and Hrp as markers of the presynaptic membrane (Eaton et al., 2002). Moreover, the key molecules for synaptic assembly of presynaptic components, such as the microtubule skeleton proteins Futsch (Keller et al., 2011; Xiong and Collins, 2012) and brp (Keller et al., 2011), are decreased in the early stage of synapse degeneration in neurodegenerative models.

Neurexins (NRXs) (Ullrich et al., 1995; Zhang et al., 2018) and Neuroligins (NLGs) (Polepalli et al., 2017; Wu et al., 2019) are synaptic cell adhesion molecules that bridge the synaptic cleft, organize molecules for synapses, mediate transsynaptic signaling, and shape neural network properties. All of these molecules show strong expression in NMJ pre- and/or postsynaptic compartments, and loss of *dnrx* and/or *dnlgs* leads to significant defects in synaptic growth, ranging from abnormal bouton size/bouton number and abnormal active zones to misformed pre- and postsynaptic structures at the *Drosophila* NMJ. However, most studies on *dnrx* and *dnlgs* focus on synapse growth and formation, but the roles of *dnrx* and *dnlgs* in synaptic degeneration are poorly understood.

Therefore, we characterized the ultrastructure of synaptic degeneration at *Drosophila* larval NMJs, including synaptic vesicles, in the *w^1118^*, *dnrx* and *dnlgs* lines as well as in pupae, and the results showed that NMJ bouton degeneration occurs in wild-type *Drosophila* and is accelerated in *dnrx* and *dnlgs* mutants.

## Materials and methods

### *Drosophila* stocks

The *w^1118^* strain was used as the wild-type control in this study. The following fly mutants were used: *dnrx^83^*, *dnrx^174^* (Zeng et al., 2007), *dnrx^273^* (Li et al., 2007), *dnlg1^ex1.9^*, *dnlg1^ex2.3^* (Banovic et al., 2010), *dnlg2^KO70^* (Sun et al., 2011), *dnlg3^KO127^* (Xing et al., 2014), and *dnlg4*^KO10^ (Zhang et al., 2017). The double mutants *dnrx^83^;dnlg3^KO127^* and *dnlg2^KO70^;dnlg3^KO127^* were generated in our laboratory. All stocks were cultured in standard medium at 25°C.

### Transmission electron microscopy analysis of larval NMJ boutons

TEM was performed according to the procedure described in our previous paper (Guangming et al., 2022; Zhou et al., 2023). In brief, wandering third-instar larvae were dissected in ice-cold disks in Jan solution (128 mM NaCl, 2 mM KCl, 4 mM MgCl_2_, 35 mM sucrose, 5 mM HEPES, pH 7.4) using standard techniques and then fixed with a mixed fixative containing 2% glutaraldehyde and 2% formaldehyde (dissolved in 0.1 M sodium cacodylate buffer, pH 7.4) at 4°C overnight. The samples were rinsed with cacodylate buffer several times at 4°C, postfixed for 2 h with 1% OsO_4_ in 0.1 M cacodylate buffer and rinsed twice with distilled water. Then, the samples were stained for 2 h with 2% saturated uranyl acetate and rinsed twice with distilled water. The specimens were dehydrated in an increasing ethanol series (30, 50, 70, 85, 95, 100% twice), passed through propylene oxide twice, and embedded into a sheet in Epon812 (SPI Science). The sheet was serially sectioned at 80 μm at the 6th/7th muscles of the A_3_ or A_2_ segment in one animal using a diamond knife on a Leica UC7 ultrathin microtome; each ultrathin slice was 90 nm thick. Approximately 30–40 slices were gathered into a group and attached to a grid, and approximately 30 grids were used in each sample. The grids were stained again with 2% saturated uranyl acetate in 50% ethanol and then with 1% lead citrate (pH 12). Finally, each ultrathin slice was examined under a transmission electron microscope (Hitachi H-7650). More than 20 wild-type animals were analyzed, and 3 animals were analyzed for each of the other strains.

### Transmission electron microscopy of pupal NMJ boutons

Pupae (13 h after pupa) were fixed to dissecting dishes with needles at both ends and covered with a few drops of fixative (2% glutaraldehyde and 2% formaldehyde in 0.1 M sodium cacodylate buffer, pH 7.4). Then, the dorsal midline was cut longitudinally with scissors without removing the internal organs. Forty minutes later, both ends of each pupa were cut to promote fixation, and the pupal samples were fixed for 24 h in fixative at 4°C. Then, the pupae were postfixed for 2 h with 1% OsO_4_, stained for 2 h with 2% saturated uranyl acetate, dehydrated in an ethanol series, passed through propylene oxide, treated with propylene oxide and epoxy resin, embedded and polymerized. Semithin slices along the side of the pupae were prepared and stained with toluidine blue to position type I boutons in NMJs, and thin sections of approximately 90 nm were prepared, collected and attached to grids. The grids were poststained with 2% saturated uranyl acetate and 1% lead citrate (pH 12) and observed under a transmission electron microscope (Hitachi H-7650).

### Transmission electron microscopy of larval and pupal ventral nerve cord

The ventral nerve cord of the late 3rd wandering instar and the pupae (6 h after pupa) were dissected in Jan solution (128 mM NaCI, 2 mM KCl, 4 mM MgCI, 35 mM sucrose, 5 mM Hepes, PH 7.4) within 20 min, and fixed in a mixture of 2% glutaraldehyde and 2% formaldehyde in 0.1 M sodium cacodylate buffer (PH 7.4) at 4°C overnight. The following experimental procedures are the same as those of TEM analysis of larval NMJ boutons.

### Pre-embedding immunogold electron microscopy procedure

Pre-embedding immunogold electron microscopy was performed as follows (Gan and Zhang, 2018). In brief, third-instar larvae were dissected in ice-cold disks in Jan solution using standard techniques and then fixed (4% formaldehyde, 0.5% glutaraldehyde, and 10% saturated picric acid in 0.1 M sodium cacodylate buffer, pH 7.4) for 4 h at 4°C (the following procedures were carried out at 4°C). The specimens were washed 4 times with 0.1 M sodium cacodylate buffer and perforated with 1% saponin for 1 h. Then, specimens were preincubated in 0.5% bovine serum albumin (BSA) and 0.1% gelatin with 0.1% saponin for 1 h and incubated with a mouse primary antibody (anti-synaptotagmin, 3H2 2D7, 1:10 DSHB; and anti-synapsin, 3C11, 1:10; DSHB) for 24 h. After 4 rinses with 0.1% Tween-20 in 0.1 M PBS, the samples were preincubated with 0.5% BSA, 0.1% gelatin and 0.1% saponin again for 1 h; incubated with a 1.4 nm ultrasmall gold-conjugated secondary antibody (goat anti-mouse IgG secondary antibody, Nanoprobes, #2001, 1:50) for 12 h; and rinsed 4 times with 0.1% Tween-20 in 0.1 M PBS. The samples were then postfixed in 2.0% glutaraldehyde in PBS for 30 min and rinsed several times with distilled water. Silver enhancement (HQS kit; Nanoprobes, #2012) was performed in a dark room for 25 min, followed by rinsing with distilled water. After rinsing with PBS for 10 min, the samples were osmicated (0.5% OsO_4_) in 0.1 M sodium cacodylate for 0.5 h. All samples were washed three times with distilled water and then stained with 2% aqueous uranyl acetate for 2 h. Subsequent gradual dehydration, epoxy resin embedding, trimming and thin sectioning were performed as described above for the NMJ boutons at the 6th/7th muscles in the A_3_ or A_2_ segment.

### Immunochemistry

Immunostaining of the larval samples was performed as described previously (Gan and Zhang, 2018). Briefly, the wandering larval samples were dissected in Jan solution, fixed in 4% paraformaldehyde at room temperature, washed with PBS and 0.3% PBST (0.3% Triton X-100 in PBS), and blocked in 1% BSA for 1 h. The samples were then incubated with anti-Hrp (Jackson ImmunoResearch, West Grove, PA), anti-synaptotagmin (1:50), and anti-synapsin (1,50) antibodies at 4°C for 2 h and then with fluorophore-conjugated secondary antibodies (Invitrogen, 1:500) for 1 h at room temperature. The samples were washed extensively with PBST and mounted in VectaShield mounting medium (Vector Laboratories). Images were collected using an Olympus FV3000 confocal microscope. During observation of degenerated NMJ boutons, the large pinhole of the confocal microscope was adjusted to increase the thickness of a single optical section, and the 3D analysis function of the confocal microscope was utilized to observe the complete NMJ bouton in the 6th/7th muscles in the A_3_ or A_2_ segment.

### Statistical analysis

The degenerate boutons in the 6th/7th muscles of A_2_–A_3_ segments from *Drosophila* larvae were counted. For each strain, at least 6 animals were analyzed and 11 segments were counted for confocal microscopy data. At least three animals or three segments per mutant strain were counted for TEM analysis, except for *dnrx*^83^, *dnrx*^174^, and *dnrx*^83/174^ which were two segments. The data were analyzed with GraphPad Prism 7 using one-way ANOVA analysis, two-tailed t tests.

## Results

### The ultrastructure of severely degenerated NMJ boutons in wild-type *Drosophila*

**Figure 1:**
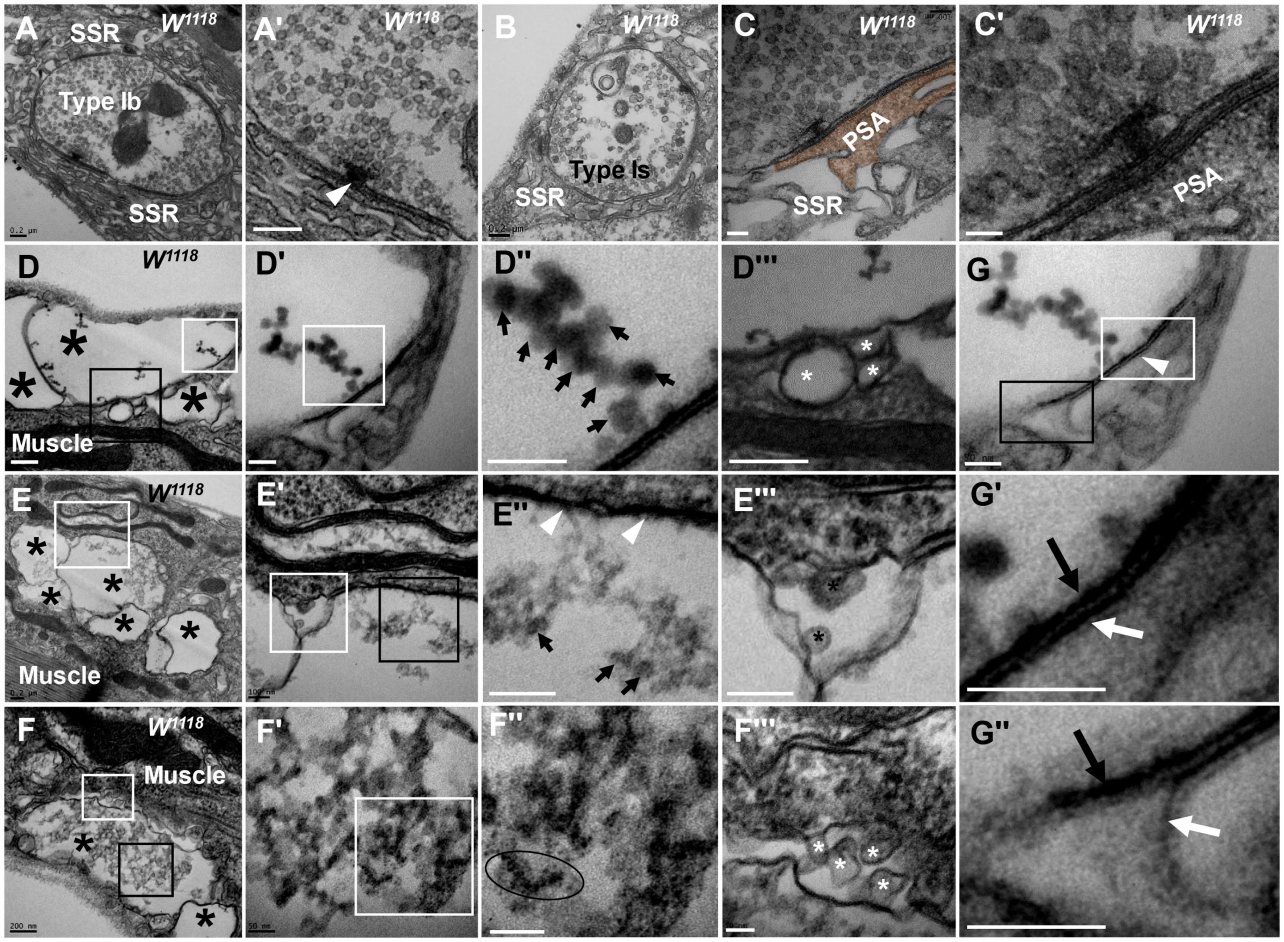
Thorough degeneration of NMJ boutons in wild-type *Drosophila* Normal type I boutons include type Ib boutons **(A)** and type Is boutons **(A’)**, with synapses and T-bars **(B,C,C’)**. Thoroughly degenerated NMJ boutons gather together and appear vacuolated, with extremely scarce SSR, in the outer **(D,D’”,F,F’”)** and inner **(E,E”)** muscles. Beaded dark synaptic vesicles overlap near a residual synapse **(D”)**. Agglomerated degenerated products with sparse dark synaptic vesicles **(E”)**. Dark ultrafine particles of less than 2 nm in the degenerating bouton **(F”)**. The residual SSR membrane in degenerated boutons **(D”’–F”’)**. A residual synapse without T-bars **(G,G”)** or a synaptic cleft. Large asterisk arrows show axon terminals, small asterisk arrows show residual SSR membrane, wedges show synapses or T-bars, black arrows show presynaptic membranes, and white arrows show postsynaptic membranes. Scale bars in **A,B**,**D**–**F**: 200 nm; **C**,**D’**,**D”**; **E’**,**E”’**: 100 nm; **C’**,**F’**,**F”**,**G**,**G”**: 50 nm.

In our early research, we analyzed a large number of type I NMJ boutons in wild-type *Drosophila* flies using TEM (Gan and Zhang, 2018). Only type I NMJ boutons between the 6th/7th muscles were presented (Atwood et al., 1993; Jia et al., 1993), and they were divided into type Ib and type Is. TEM revealed that the type Ib bouton (large) (Figure 1A) was globular, the regular dense SSR membrane circled around the axon terminal, and clear synaptic vesicles gathered in a T-bar (Figures 1A,A’) before the presynaptic membrane with an obvious synaptic cleft. Type Is boutons are smaller, and the SSR membrane is thinner (Figure 1B; Atwood et al., 1993; Jia et al., 1993). In addition, there was a compact postsynaptic area (PSA) that matched after the postsynaptic membrane in both type I boutons (Figures 1C,C’; Chen et al., 2012) and type Ib boutons. In the type I bouton terminal, organelles, such as mitochondria (Figures 1A,B), were also present.

However, analysis of more than 20 larvae showed that degenerated NMJ boutons were rarely observed in wild-type *Drosophila*. Severely degenerated NMJ boutons showed extremely degenerated axon terminals and extremely severe retraction of the SSR. The degenerated terminals gathered in a small area of the outer muscle (Figure 1D) with rare retracted SSR membranes (Figures 1D,D’’’), and they were basically vacuolated (Figure 1D) or contained beaded, overlapping, dark synaptic vesicles (Figures 1D’,D’’) and residual synapses (Figures 1G,G’’) without mitochondria or T-bars. Compared to presynaptic and postsynaptic membranes in normal synapses with a typical T-bar structure (Figures 1A’,C’), the residual synapse had a significantly thin presynaptic membrane and postsynaptic membrane that were stuck to each other, with almost no synaptic cleft (Figures 1G,G’’). The residual presynaptic and postsynaptic membranes were thin and dark without synaptic clefts or T-bars (Figures 1E’’,G,G’’) and sometimes had a tendency to separate from each other (Figures 1G,G’’), which showed the characteristics of complete synapse degeneration.

The degenerated NMJ boutons were also present inside muscle (Figures 1E,E’’’); some were completely vacuolated (Figure 1E), but some vacuolated boutons contained degenerated ultrafine particles and had a sparse profile of similar dark synaptic vesicles (Figure 1E’’). Furthermore, larger degenerated NMJ boutons in the outer muscle were filled with agglomerated degenerated products (Figure 1F) and did not contain clear and dark synaptic vesicles but rather dark ultrafine particles less than 3 nm in diameter (Figures 1F’,F’’). The SSR was extremely retracted in all degenerated NMJ boutons (Figures 1D’’’–F’’’), and residual SSRs were rare. There was no T-bar structure or mitochondria in any of the degenerated NMJ boutons.

In severely degenerated NMJ boutons in wild-type *Drosophila*, the axonal terminal retracted and shrank, with retraction and degradation of presynaptic components, such as synaptic vesicles, mitochondria and the T-bar structure, and most SSR membranes were retracted from the NMJ boutons. It is worth noting that normal NMJs were singular and isolated by an SSR (Figures 1A,B), while the severely degenerated NMJs were close together without an obvious SSR (Figures 1D,F,F’).

During the pre-pupa, the synaptic terminals would be engulfed by glial cells (Tasdemir-Yilmaz and Freeman, 2014). These engulfed synaptic terminals also have completely clear vesicles, some dark vesicles, and all dark vesicles (Data not displayed).

### Gradual degeneration process of NMJ boutons in wild-type *Drosophila*

**Figure 2:**
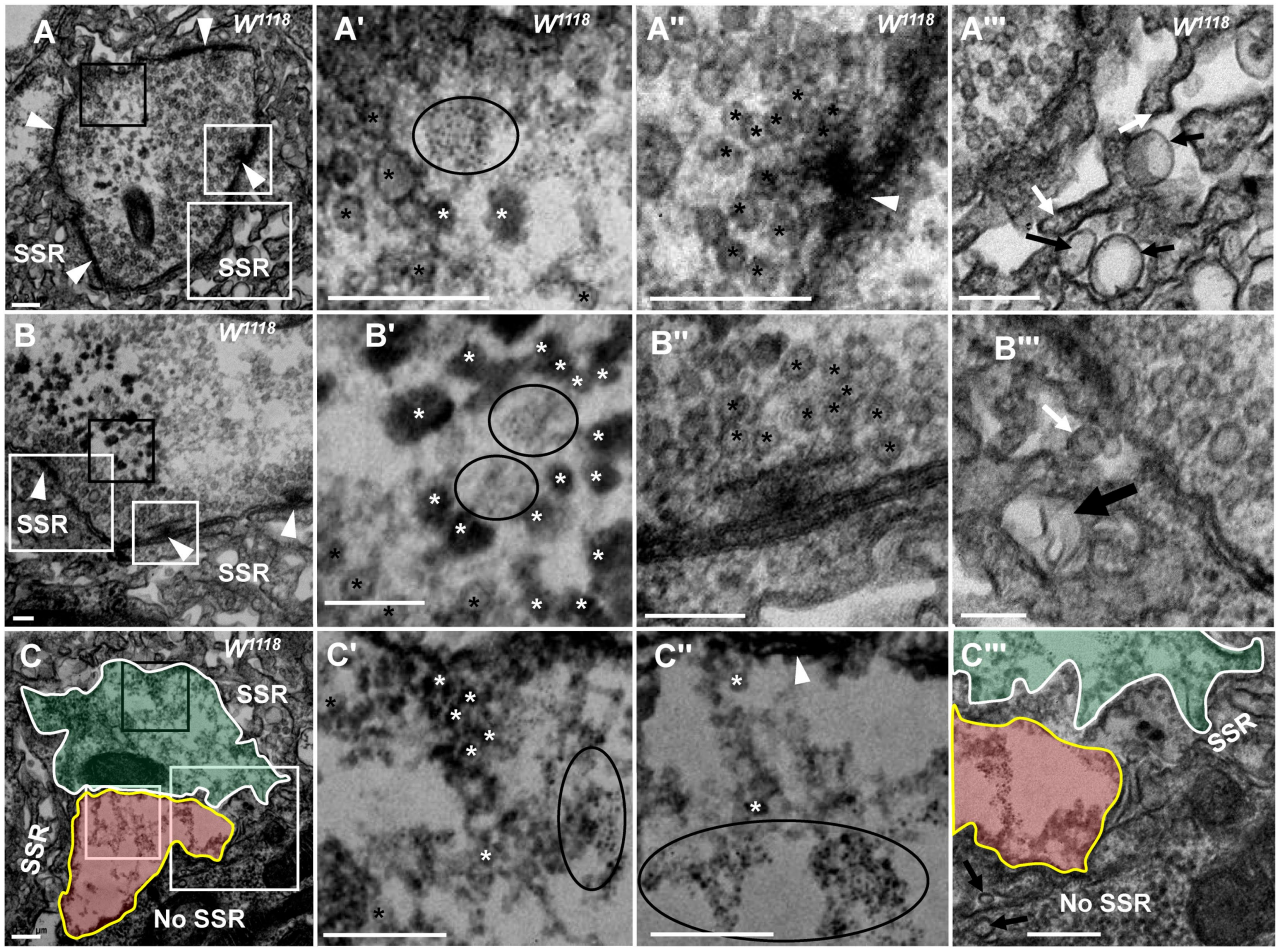
NMJ bouton is gradually degenerated in synaptic vesicles and SSR Fewer dark synaptic vesicles and dark ultrafine particles and more typical clear synaptic vesicles **(A,A’)** in a mildly degenerated bouton with a typical T-bar **(A,A”)**. More dark synaptic vesicles and dark ultrafine particles and fewer typical clear synaptic vesicles **(B,B’)** in a moderately degenerated bouton with a typical T-bar **(B,B”)**. Extremely rare clear synaptic vesicles, several dark synaptic vesicles, and many dark ultrafine particles **(C,C’,C”)** in a severely degenerated bouton without a T-bar structure **(C)**. SSR membranes start withdrawing and swelling in a mildly degenerated bouton **(A”’)**, fold inward in a moderately degenerated bouton **(B”’)**, and become more disordered, looser or even missing in a severely degenerated bouton **(C”’)**. Black asterisk arrows show typical clear synaptic vesicles, white asterisk arrows show dark synaptic vesicles, and ellipses show dark ultrafine particles. White arrows show membrane withdrawal, the thin black arrow shows membrane swelling, and the thick black arrow shows membrane folding. Wedges show synapses or T-bars. The curves in panel **(C)** show two adjacent degenerated boutons, and the curve in panel **C”’** shows a disordered and thin SSR membrane. **A’**–**C’** are enlargements of the black boxes in **A**–**C**, respectively. **A”**–**C”** are enlargements of the small white boxes in **A**–**C**, respectively. **A”’**–**C”’** are enlargements of the large white boxes in A, B, and C, respectively. Scale bars in **A**,**A”’**, **C**,**C”’**: 200 nm; **B**,**B”’**:100 nm.

We observed and confirmed severely degenerated NMJ boutons with full retraction into small boutons and then identified the process of degeneration in wild-type *Drosophila* (Figures 2A–C).

In the milder degeneration state (Figures 2A,A’’’), the boutons looked similar to normal globular type Ib boutons, but in the center region of the terminal, several dark synaptic vesicles and dark ultrafine particles (Figures 2A,A’) appeared with clearer synaptic vesicles (Figure 2A’) and a normal T-bar (Figure 2A’’). Furthermore, the SSR membrane swelled and withdrew (Figure 2A’’’). In a moderately degenerating bouton (Figures 2B,B’’’), the dark synaptic vesicles and dark ultrafine particles increased (Figure 2B’), and clear synaptic vesicles were present around the normal T-bar (Figure 2B’’). However, the SSR membrane further loosened and withdrew, and some swollen SSR membranes folded inward (Figure 2B’’’). Then, two severely degenerated type Ib boutons were observed, which were close to each other without a T-bar (Figure 2C). The two boutons had irregular terminals (Figures 2C,C’’’) in which there were many dark ultrafine particles (Figure 2C’’), few dark synaptic vesicles (Figure 2C’) and few clear synaptic vesicles (Figure 2C’’). The SSR became disordered and collapsed, and the SSR membrane was loose and thin or even absent from some regions of severely degenerated boutons with extremely sparse residual SSR membranes (Figure 2C’’’). Dark synaptic vesicles appeared to be the intermediates, and the dark ultrafine particles were the final product during the collapse and degeneration of clear synaptic vesicles. It is likely that in TEM, severely degenerated boutons are different from synaptic footprints that contain a relatively complete SSR and postsynaptic Dlg protein but no synapsin or Hrp (Eaton et al., 2002; Eaton and Davis, 2005). It is worth noting that the deformed axon terminal was not detached from the SSR of degenerated boutons but instead degenerated *in situ*.

**Figure 3:**
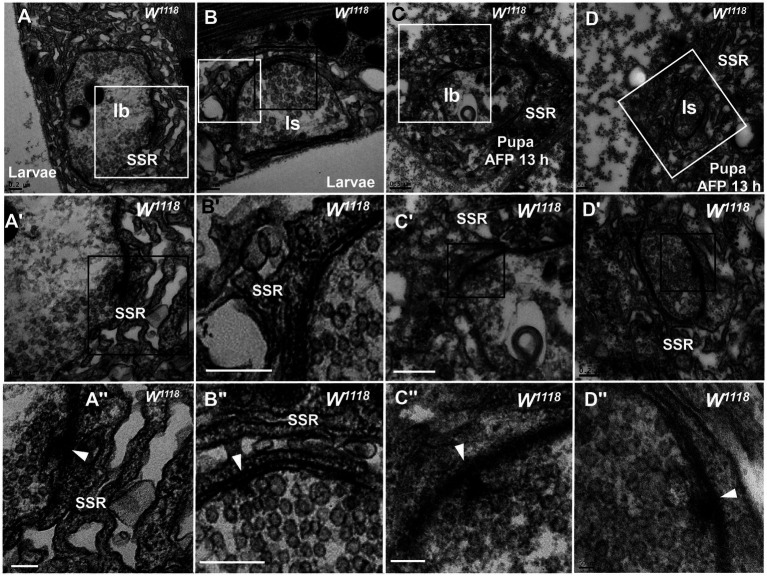
The initiation site of NMJ bouton degeneration Swollen and loose SSR membranes with typical clear vesicles and T-bars are shown in a type Ib bouton **(A,A”)** and type Is bouton **(B,B”)** of the third-larval stage. Obviously swollen and loose SSR membranes with typical clear vesicles and T-bars are shown in a type Ib bouton **(C,C”)** and a type Is bouton **(D,D”)** in the pupa stage. Wedges show the synapse or T-bar. **A’**–**D’** are enlargements of the white boxes in **A–D**, respectively; **A”–D”** are enlargements of the black boxes in **A’–D’**, respectively. Scale bars in **A**,**B**,**B”**, **A”**,**B**,**D**,**D’**: 200 nm; **A**,**A’**,**C’**: 500 nm; **D”**: 50 nm.

Degeneration of NMJ boutons originated from SSR abnormalities in the wild-type fly. The SSR membrane, synaptic vesicles, and T-bar showed marked degeneration in boutons (Figures 1, 2), but which component was the first to become abnormal remains unknown. The SSR membrane became loose and swollen in type Ib boutons (Figures 3A,A’’) and type Is boutons (Figures 3B,B’’), but the synaptic vesicles and T-bars were very typical in both types of boutons (Figures 3A’’,B’’), and the center region of the boutons did not exhibit degeneration, as shown in Figures 2A–C. Moreover, there were no dark synaptic vesicles or dark ultrafine particles in type Ib or type Is boutons (Figures 3A,B). NMJ boutons undergo marked degeneration during the process of development in the early pupal stage (6 h pupa) (Liu et al., 2010). We found that the T-bars were typical and that most synaptic vesicles were clear and normal in both type Ib boutons (Figures 3C,C’’) and type Is boutons (Figures 3D,D’’), but the SSR membrane was obviously swollen, thin, loose and disordered (Figures 3C,C’,D,D’’) in the pupal stage (13 h pupa). Furthermore, there were no dark synaptic vesicles or dark ultrafine particles in type Ib or type Is boutons in the early pupal stage (Figures 3C,D).

Therefore, degeneration of NMJ boutons originates from swelling and retraction of the SSR membrane, and the clear synaptic vesicles then turn into dark synaptic vesicles and fragment into dark ultrafine particles along with degeneration of the T-bar structure from the presynaptic membrane.

### *dnrx* mutation leads to NMJ boutons degeneration

**Figure 4:**
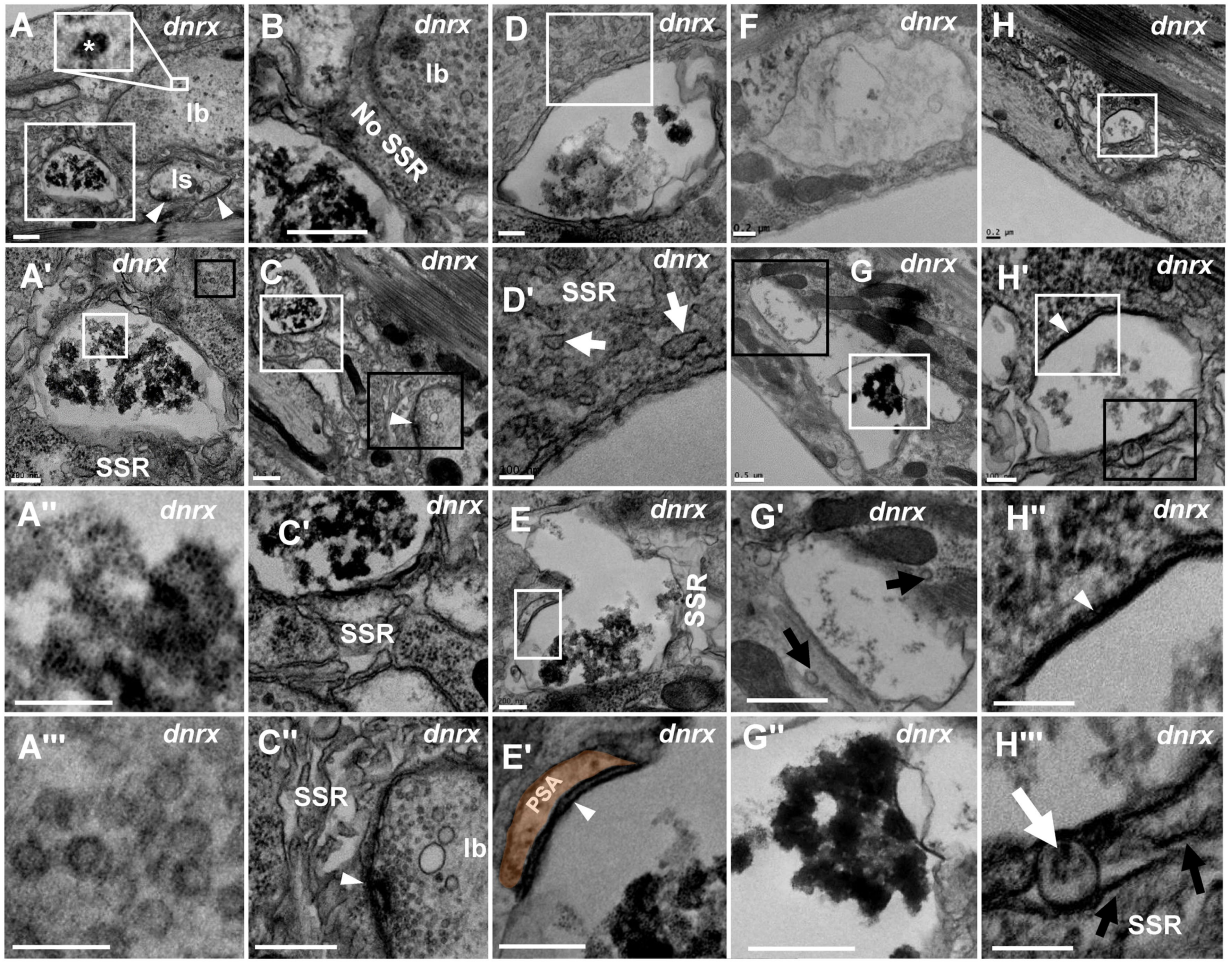
NMJ bouton is severely degenerated in the *dnrx* mutant A normal type Ib bouton **(A)** is adjacent to a degenerated bouton with obviously agglomerated dark ultrafine particles **(A’,A”)** filled with dense clear vesicles **(A’,A”’)**. There is no SSR between the normal bouton and the degenerated bouton **(B)**. The retracted SSR membrane near the degenerated bouton becomes obvious **(C,C’)**. The SSR membrane is relatively intact near the type Ib bouton with a T-bar and clear vesicles **(C,C”)**. The seriously degenerated bouton has swollen, discontinuous remnants of the SSR membrane **(D,D’)**. There are linear SSR remnants **(E,E’,E”)** in a degenerated bouton. The irregular appearance of degenerated ghost boutons (**F–H”**). Sparse **(F,G’)** and agglomerated **(G,G”)** dark ultrafine particles in the axon terminal and thin SSR membrane near the ghost bouton **(G’)**. Degeneration occurs in a developing bouton in which there are dark ultrafine particles **(H,H’)**, completely degenerated synapses **(H,H”)**, and retracted and linear SSR membranes **(H,H”)**. Wedges show synapses or T-bars. The curves in panel **E’** show shrinking PSA. White arrows show swelling of the SSR membrane, and black arrows show retraction of the SSR membrane. **A’**,**A**”,**C-E’**,**G’**,**H’**,**H”** are enlargements of the white boxes in **A**,**A’**,**C**–**E**,**G**,**H**, and **H’**, respectively. **A”’**, **G”**, **H’** are enlargements of the black boxes in **A’**,**G**, **H’**, respectively. Scale bars in **A**–**C**,**C”**,**G**,**G”**: 500 nm; **A’**,**D**,**E**,**E”**,**F**,**H**: 200 nm; **A”**,**A”’**,**D’**,**H’**-**H”’**: 100 nm.

**Figure 5:**
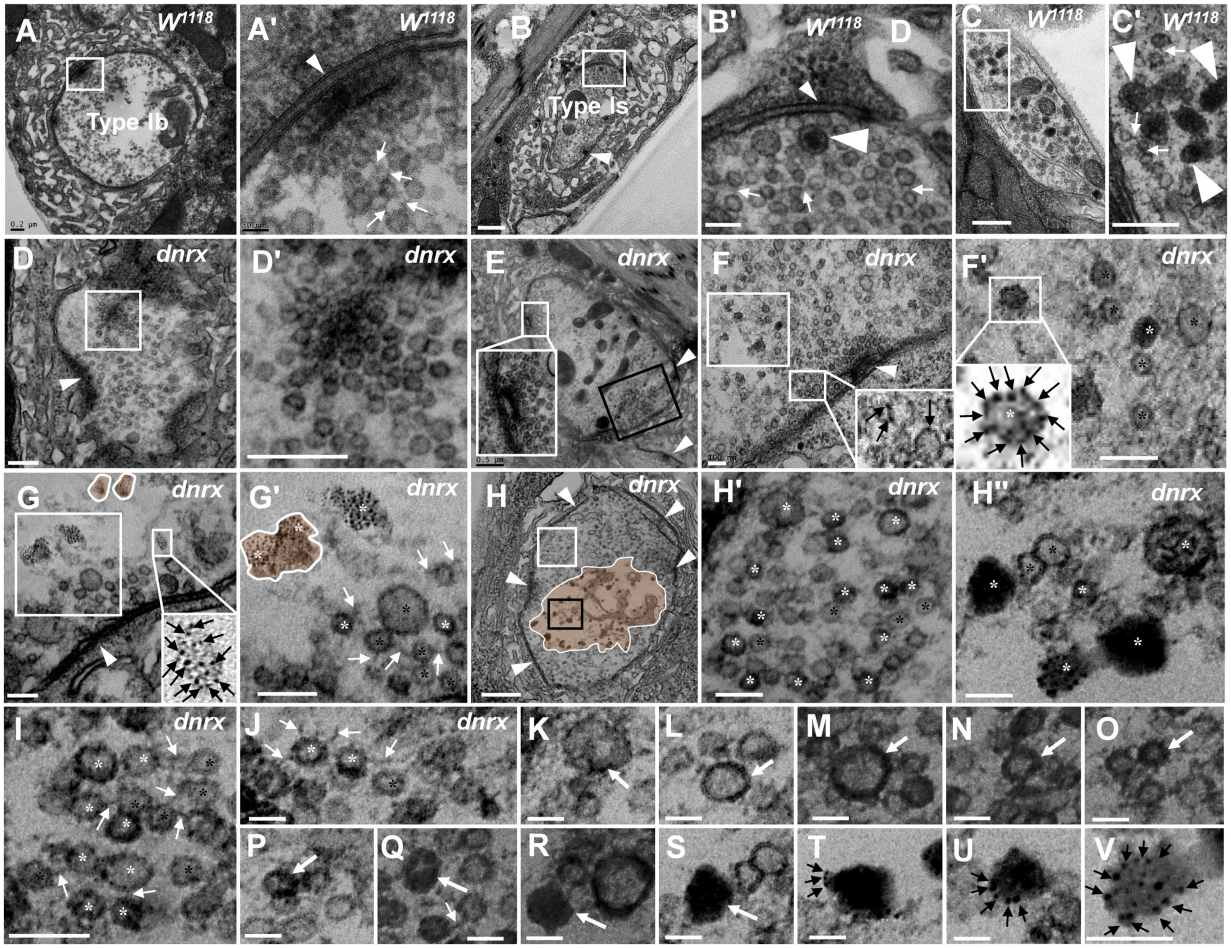
The processes of NMJ bouton degeneration in *dnrx* The clear synaptic vesicles and T-bar in a type Ib bouton **(A,A’)**, dense core vesicles in a type Is bouton **(B,B’)** and type II bouton **(C,C’)**; most clear synaptic vesicles were clustered with actin filament in wild-type **(A’,B’,C’)**. The T-bar **(D)** detaches from the presynaptic membrane and floats in the terminal region with dense synaptic vesicles **(D’)**. T-bar liked to detach, clusters of dense synaptic vesicles before the presynaptic membrane **(E)**, and some synaptic vesicles in the same bouton deviate from the presynaptic membrane (**E**, black box). The first mode of synaptic vesicle degeneration **(F,G’)**. The synaptic vesicles degenerate at the membrane without actin filaments **(F,F’)**. The ultrafine spots dispersed into ellipses **(G)** or irregular profiles with dark ultrafine particles **(G’)**. The second mode of synaptic vesicle degeneration **(H–V)**. The degenerating type Ib bouton without a T-bar **(H)** was full with clear vesicles and vesicles of different densities **(H’)** in the cortex of the axon terminal **(H,H’)**, and clear vesicles, dense vesicles, dark clumps without a membrane and dark ultrafine particles were distributed in the center of the terminal **(H,H”)**. Clear vesicles and vesicles of different densities are clustered with actin filaments near the presynaptic membrane **(I)** or detach from each other without actin filaments **(J)**. A short dark linear occurs **(K)** and expands **(L)** to the whole membrane **(M,N)**, and the electron density gradually expands inward towards vesicles **(O,P,Q)**. The vesicle is full and electron dense without membrane or actin filament **(R)**. The darker clump **(S)** separates several dark ultrafine particles at the edge of the clump **(T)**. The ultrafine particles increase at the edge of the clump **(U)** until many dark ultrafine particles appear **(V)**. Small wedges show synapses or T-bars, large wedges show dense core vesicles, black arrows show ultrafine particles, thin white arrows show filaments, thick white arrows show the process of vesicle degeneration with the second mode of degeneration, the curves in panel **G,G’** show degenerated vesicles with the first mode of degeneration, and the curves in panel H show that degeneration occurs in the center of the terminal. Black asterisk arrows show typical clear synaptic vesicles, white asterisk arrows show vesicles of different densities, dark clumps or gathered dark ultrafine particles. **A’**–**H’** are enlargements of the white boxes in **A–H**, respectively; **H”** is an enlargement of the black box in **H**, and **I–V** are all enlarged from **H**. Scale bars in **A**, **C,C’**, **D**,**D’**: 200 nm; **B**,**H**: 500 nm; **A’**, **H’**,**H”**, **J–V**: 50 nm; **B’**, **F**,**F’**,**G**,**G’**, **I**: 100 nm.

We analyzed *dnrx*, *dnlg1*, *dnlg2*, *dnlg3* and *dnlg4* single mutants and found that *dnrx^273^*, the nrx null mutant, led to degeneration of NMJ boutons in *Drosophila*. The degenerating NMJ boutons in *dnrx*^273^ flies (Figures 4, 5) demonstrated more significant degeneration than those in wild-type *Drosophila* (Figures 1–3). The terminal of degenerated NMJ boutons, without a T-bar structure or other organelles, was smaller than that of normal boutons (Figure 4A) and was filled with dark ultrafine particles (Figures 4A’,A’’) but lacked dark synaptic vesicles, whereas the adjacent type Ib bouton was filled with clear vesicles (Figures 4A’,A’’) and several dark synaptic vesicles (Figure 4A). There was no SSR membrane between the degenerated bouton and the adjacent normal bouton (Figure 4B), which also suggested that the SSR retracted severely as the NMJ bouton degenerated. The SSR membrane was sparse and loose near the degenerated bouton (Figures 4C,C’) but was relatively normal compared with that of the adjacent type Ib bouton (Figures 4C,C’’). In the seriously degenerated boutons, the axon terminals showed signs of degeneration/vacuolization, and the SSR membrane was obviously swollen and withdrawn (Figures 4A’,A’’,C’,D,D’). It is worth noting that the contents of degenerated terminals in *dnrx*^273^ mutants (Figures 4A’,C’,D,E) were much denser than those of wild-type terminals (Figures 2C,C’’). We observed another degenerated bouton that had an irregular morphology, a seriously linearized and degenerated SSR (Figures 4E,E”), degenerated contents in the axon terminal, an obvious residual postsynaptic area (PSA) and a synapse with almost no synaptic cleft (Figures 4E,E’). The residual PSA suggested that the NMJ bouton was degenerated *in situ* but not eliminated. The degeneration of NMJ boutons could originate from ghost and developing boutons. The abnormal SSR phenotype was not observed in ghost synapses due to their lack of an SSR. The appearance of large ghost boutons was irregular in *dnrx^273^* mutants (Figures 4F,G), while normal ghost boutons in wild-type flies (Gan and Zhang, 2018) and in some mutants (Packard et al., 2002) were spherical and full of clear vesicles. Instead of the clear and dark synaptic vesicles, dense dark ultrafine particles were observed in the ghosts (Figures 4F,G). However, some dark ultrafine particles were sparse (Figures 4F,G’), and other dark ultrafine particles were intensively clustered (Figures 4G,G’’). A thin SSR membrane was occasionally visible (Figure 4G’). The degeneration might originate from developing boutons in which there were dark ultrafine particles (Figures 4H,H’), degraded synapses with thin presynaptic and postsynaptic membranes (Figures 4H,H’’), and swollen and linear SSR membranes (Figures 4H’,H’’’).

Since mutant NMJ bouton degeneration was more serious in the *dnrx^273^* mutant, we suspected that it might be easier to observe the fine degeneration of synaptic vesicles in the mutant by utilizing TEM. As reported in the literature (Atwood et al., 1993; Jia et al., 1993), type Ib boutons had clear synaptic vesicles with a T-bar (Figures 5A,A’), type Is had extremely sparse dense core vesicles (Figures 5B,B’), type II had more dense core vesicles (Figures 5C,C’’), type III included only dense core vesicles (data not shown), and most clear synaptic vesicles were clustered with actin filaments (Figures 5A’–C’).

Before NMJ boutons were severely degenerated, the T-bars detached from the presynaptic membrane. In all degenerated boutons, we observed a residual synapse that included only the presynaptic and postsynaptic membranes, but no presynaptic T-bar that recruits and docks synaptic vesicles was present. The T-bar (Figure 5D) detached from the presynaptic membrane, and the shed T-bar clustered dense synaptic vesicles (Figures 5D,D’; Aberle et al., 2002), which might have hindered accumulation of synaptic vesicles near the presynaptic membrane (Figures 5D’,E’ (shown in the black box)) and moved them to the center of the NMJ bouton. However, in the same type Ib bouton, the peripheral synaptic vesicles gathered in another T-bar that looked as if it was about to detach from the presynaptic membrane.

Then, we observed two degeneration modes of synaptic vesicles in type Ib boutons that could avoid the interference of dense core vesicles in type Is, type II and type III, according to the electron density under electron microscopy. In the first mode, one or two dark ultrafine spots occurred on the membrane of clear synaptic vesicles near a synapse with a relatively intact T-bar (Figure 5F), and more dark ultrafine spots developed on the clear synaptic vesicle membrane and formed a circle at a site farther from the same synapse (Figures 5F,F’). Before another synapse without a T-bar, the dark ultrafine spots dispersed into irregular profiles with larger sizes than the other synaptic vesicles (Figure 5G’); therefore, we believe the irregular profiles were the result of collapse and dispersion from the degenerated synaptic vesicles with dark ultrafine spots, and two collapsed synaptic vesicles overlapped each other to form a large profile (Figures 5G,G’). The slightly collapsed vesicles had the appearance of an ellipsoid profile, with dark spots on the inside and outside and a size similar to that of clear vesicles (Figure 5G, lower right corner). It is worth noting that the clear synaptic vesicles away from the synapse had a tendency to detach from each other without actin filaments (Figures 5F’,G’). Therefore, the first mode of synaptic vesicle degeneration occurred on the membrane with ultrafine spots and showed a collapsed and dispersed irregular profile with dark ultrafine particles.

In the second mode, the clear synaptic vesicles degenerated into dense synaptic vesicles, formed irregular dark clumps, and collapsed and dispersed an irregular profile with dark ultrafine particles. The degenerating bouton, a type Ib bouton in the *dnrx^273^* mutant with only clear synaptic vesicles (Atwood et al., 1993; Jia et al., 1993; Figure 5H), had five synapses without T-bars and numerous synaptic vesicles on its periphery. In high magnification mode, the synaptic vesicles could be divided into clear vesicles and vesicles of different densities, both with membranes near the periphery and cortex of an axon terminal (Figures 5H,H’); however, clear vesicles, dense vesicles, dark clumps without a membrane, and dark ultrafine particles were present in the center of the axon terminal (Figures 5H,H”). The dense and clear vesicles could be clustered with actin filaments (Figure 5I) or detached without actin filaments (Figure 5J), and they appeared to exhibit deepening electron density (Figures 5K–V). A short, dark line occurred on a certain point on the clear vesicle membrane (Figure 5K), and the dark line expanded along the synaptic vesicle membrane (Figure 5L) until it was completely covered (Figures 5M,N), which made the vesicle dark. The electron density in vesicles expanded inward (Figure 5O), and the clear region in the dark vesicle continually decreased (Figures 5P,Q). Then, the vesicle became fully electron dense, the membrane and morphological profile of the vesicle were lost, and a dark clump without actin filaments formed (Figure 5R). The clump became darker (Figure 5S) and separated into several dark ultrafine particles at the edge of the clump (Figure 5T), and the number of ultrafine particles increased at the edge of the clump (Figure 5U) until many fragmented dark ultrafine particles were present (Figure 5V), which could be regarded as direct evidence that the dark vesicle had broken into ultrafine particles. The dark clumps had different sizes due to the different vesicle sizes. Once many vesicles adhered to each other with actin filaments and degenerated together, they formed a large clump of ultrafine particles (Figures 4A’C’E’G’’). Accordingly, it was easier to observe the dynamics of synaptic vesicle degeneration in *dnrx^273^* mutants, and the speckled membranes of clear vesicles, dark vesicles, dark clumps, and dark ultrafine particles could be regarded as signs of synaptic vesicle degeneration without lysosome involvement.

### *dnlg1* and *dnlg4* mutants exhibited NMJ boutons degeneration

**Figure 6:**
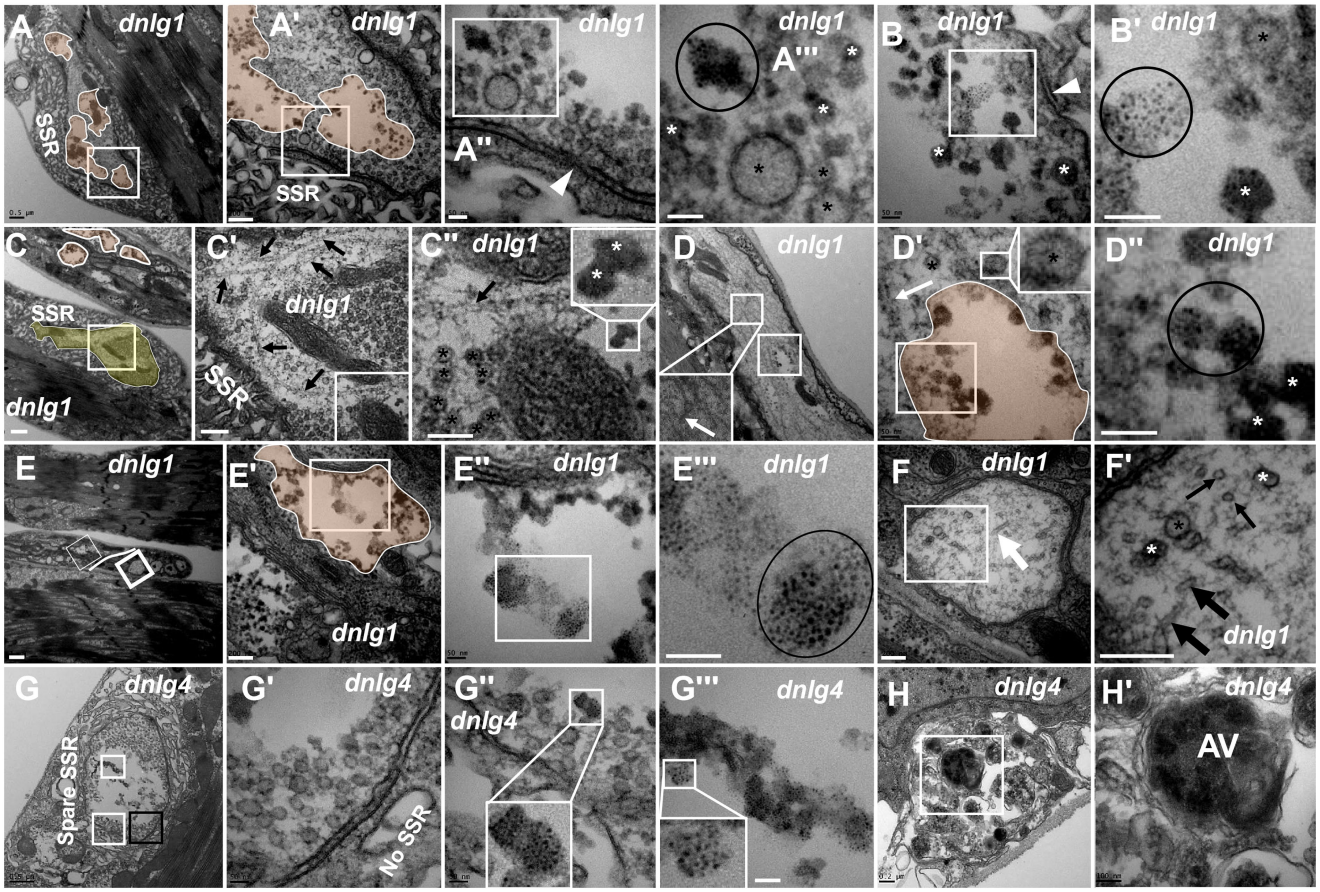
*dnlg1* and *dnlg4* mutants lead to NMJ bouton degeneration The degenerated axon terminal has several plaques in a degenerating type Ib bouton with a relatively intact SSR **(A)**. Dark synaptic vesicles and dark ultrafine particles scatter in the plaques of the degenerating axon terminal **(A’,A”’,B,B’)**. Long microtubules protruded into a degenerating type Ib bouton in the above and below directions **(C,C’)**, and the below microtubules passed through dense clear vesicles **(C’,C”)** with dark synaptic vesicles **(C”)**. White plaques occur in a large axon with different directions of microtubules **(D)** and contain dark synaptic vesicles and dark ultrafine particles **(D’,D”)**, and clear vesicles are out of the white plaque **(D’)**. Degenerated axons **(E)** are filled with dark ultrafine particles **(6E’,E”’)**, and the other axons are intact with clear vesicles and microtubules in different directions **(E,F,F’)**. The degenerated type Ib bouton is shown with a retracted SSR, dark vesicles **(G,G’)** and dark ultrafine particles **(G,G”,G”’)** in *dnlg4*. The autophagic vacuole (AV) is shown in a degenerated axon **(H,H’)**. Wedges show synapses or T-bars, white arrows show bent microtubules, thin black arrows show microtubule transection, and thick black arrows show non-transverse microtubules. The curves in panel **C** show degenerating terminals, and ellipses show clumps of ultrafine particles. Black asterisk arrows show clear synaptic vesicles, white asterisk arrows show different dark synaptic vesicles. **A’**–**A”’**,**B’**,**C’**,**C”**,**D’**,**D”**,**E’**–**E”’**,**F’**,**G’**–**G”’** and **H’** are enlargements of the white boxes in **A–A”**,**B**,C–C”,**D**,**D’**,**E–E”**,**F**,**G–G”**, and **H**, respectively. Scale bars in **A**,**C**,**D**,**G**: 500 nm; **A’**,**C’**,**E’**,**F**,**F’**,**H**: 200 nm; **A”**,**A”**,**B**,**B’**,**D’**,**D”**,**E”**,**E”’**,**G’**,**G”**: 50 nm; **C”**,**H’**: 100 nm; **E**: 1000 nm.

According to the signs of synaptic vesicle degeneration, *dnlg1* and *dnlg4* mutants exhibited NMJ bouton degeneration. There was significant degeneration of axon terminals in NMJ boutons in *dnlg1* mutants, but the SSR remained relatively intact (Figures 6A,A’,C,C’). In the axon terminal, there were several plaques (Figures 6A,A’), and the degenerated synaptic vesicles were in the plaques in the form of dark synaptic vesicles (Figures 6A’,A’’’,B,B’) and dark ultrafine particles (Figures 6B,B’) along with clear synaptic vesicles and T-bars (Figure 6A’’). Therefore, plaques with dark synaptic vesicles and dark ultrafine particles are potential markers of synaptic degeneration.

Degeneration was accompanied by abnormal assembly of microtubules. Long microtubules protruding into a type Ib bouton in both directions (from above and below) were observed (Figures 6C,C’), and the downward pointing microtubule passed through clear vesicles (Figures 6C’,C’’) and reached a small plaque with a dark synaptic vesicle that showed degeneration (Figure 6C’’). Degeneration could occur in axons. White plaques occurred among microtubules in a large axon (Figure 6D) and contained dark synaptic vesicles and dark ultrafine particles (Figures 6D’,D’’), but clear vesicles were not present in the white plaque (Figure 6D’, upper right corner). Furthermore, some axons of motor nerve fibers also showed degeneration in *dnlg1* mutants (Figures 6C,E). The degenerated axons contained dark ultrafine particles (Figures 6E’,E’’’) and gathered in specific parts of fibers (Figure 6E). In the other part of the same fiber, the axon looked intact, with clear vesicles and microtubules pointing in different directions (Figures 6E,F,F’). Therefore, the boundary between the degenerated axons and the normal axons could be artificially drawn (Figure 6E).

The axon terminals also degenerated with SSR retraction (Figure 6G) in NMJ boutons in *dnlg4* mutants, and dark vesicles (Figures 6G,G’), dark ultrafine particles and spare SSR membranes were observed (Figures 6G,G’’,G’’’). Interestingly, autophagic vacuole (AV) was found in degenerated axons (Figures 6H,H’).

### *dnlg2* and *dnlg3* coregulate synaptic degeneration in *Drosophila* NMJs

**Figure 7:**
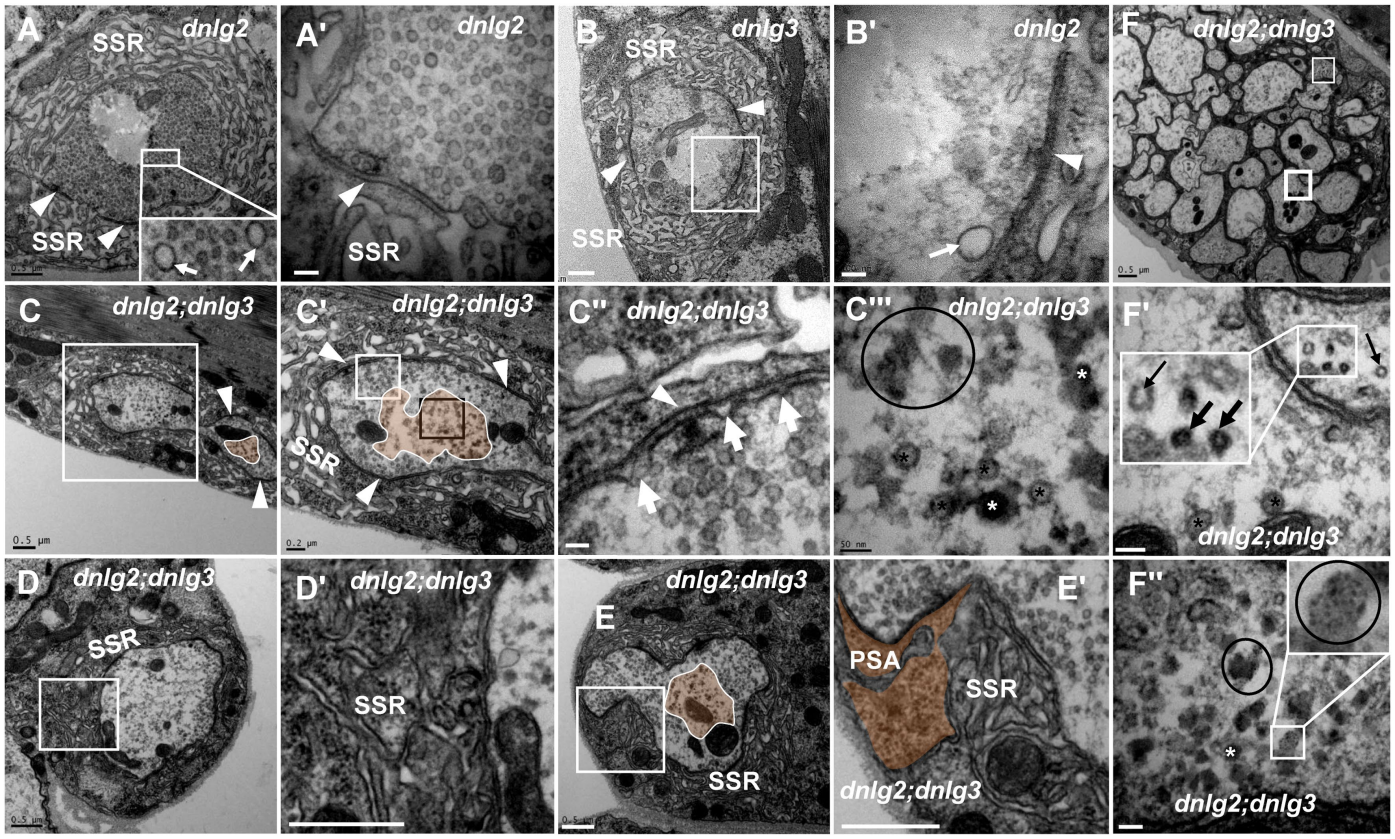
*dnlg2* and *dnlg3* coregulate degeneration in *Drosophila* NMJ There is no NMJ bouton degeneration in *dnlg2* **(A,A’)** or *dnlg3* **(B,B’)**. In the *dnlg2;dnlg3* double mutant, the terminal has a typical T-bar with ruffles of the presynaptic membrane **(C,C”)** and dark vesicles in the center **(C–C”’)**; the SSR becomes disordered **(D,D’)** and retracts to form a large PSA **(E,E’)**. The degeneration axons emerge outward from the fiber with dark vesicles and dark ultrafine particles **(F,F”)**. Inside the fiber, the periphery of the axon is relatively intact with clear vesicles **(F,F’)**, but most inside axons are disordered without dark vesicles or dark ultrafine particles **(F)**. Wedges show synapses or T-bars, white arrows show presynaptic ruffles, thin black arrows show transection of normal microtubules, and thick black arrows show transection of degraded microtubules. The curves in panel C show degenerating terminals, and ellipses show clumps of ultrafine particles. Black asterisk arrows show clear synaptic vesicles, white asterisk arrows show different dark synaptic vesicles. **A’–C’**,**C”**,**D’**, and E’ are enlargements of the white boxes in **A–C**,**C’**,**D**, and **E**, respectively. **C”’** is an enlargement of the black box in **C’**. **F’** and **F”** are enlargements of the thick and thin white boxes in **F**, respectively. Scale bars in **A–C**, **D,D’**, **E,E’**, **F**: 500 nm; **A’–C’**: 200 nm; **C”,C”’**,**F’**,**F”**: 50 nm.

Both *dnlg2* (Sun et al., 2011) and *dnlg3* (Xing et al., 2014) regulate the circulation of synaptic vesicles, but dark vesicles and dark ultrafine particles were not observed in more than 30 NMJ boutons in *dnlg2* (Figures 7A,A’) and *dnlg3* (Figures 7B,B’) single mutants or *dnlg2-* and *dnlg3-*overexpressing lines (data not shown). However, degeneration of NMJ boutons frequently occurred in both the axon terminal and SSR in *dnlg2;dnlg3* double mutants. The degeneration primarily emerged in the center of the axonal terminal (Figures 7C,C’,C’’’,E), with dark vesicles and dark ultrafine particles visible (Figure 7C’’’), but the clear synaptic vesicles were mainly distributed around the axonal membrane with presynaptic ruffles (Figure 7C’’). Moreover, the SSR was disordered (Figures 7D,D’) or even retracted to form a large PSA with the T-bar (Figures 7E,E’). In outer motor nerve fibers, the degeneration of axons mainly emerged with dark vesicles and dark ultrafine particles (Figures 7F,F’’). However, in most axons of the same nerve fiber, the periphery of the axons was relatively intact, with clear vesicles inside (Figures 7F,F’), and most axons inside the fiber (Figure 7F) looked ordered, without dark vesicles or dark ultrafine particles, which suggested that the peripheral axons of nerve fibers were more susceptible to degeneration. Interestingly, we found normal microtubules along with abnormal microtubules that had a smaller diameter and dark electron density in the small axon (Figure 7F’).

### *dnrx* and *dnlg3* co-lead degeneration in *Drosophila* NMJs

**Figure 8:**
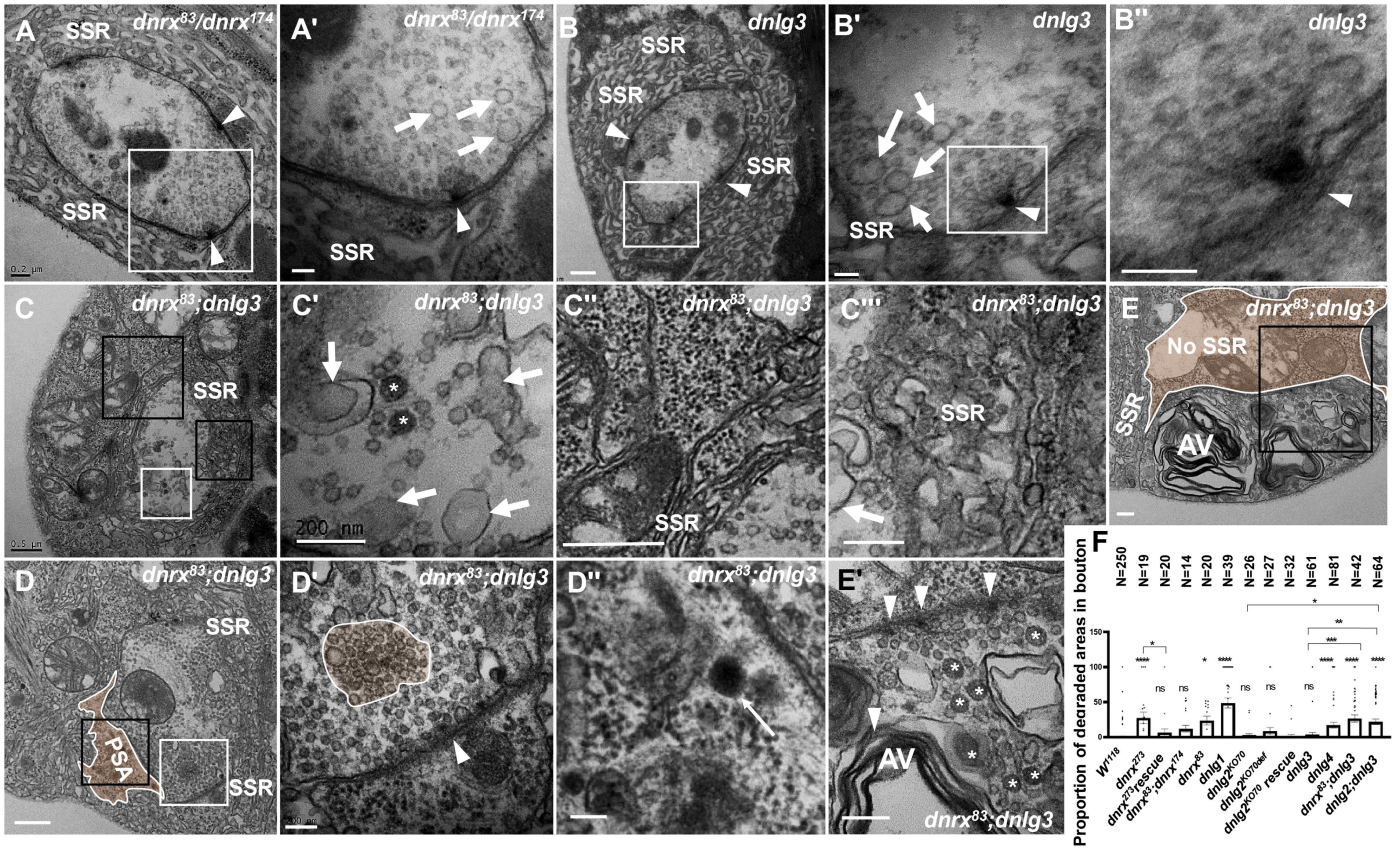
*dnrx* and *dnlg3* colead degeneration in *Drosophila* NMJ There is no NMJ bouton degeneration in *dnrx^83^/dnrx^174^* **(A,A’)** or *dnlg3* **(B,B”)**. In the *dnrx83;dnlg3* double mutant, the terminal has dark vesicles, rare clear vesicles, large clear endosomes **(C,C’)**, the SSR retracts to form a rare SSR membrane and a large PSA **(C”,D)**, and the dense SSR is disordered **(C”’)**. The T-bar detaches from the presynaptic membrane **(D,D’)**, and a lysosome is shown in SPA **(D,D”)**. A degenerated type Is bouton contains a T-bar, dark vesicles **(E,E’)**, and myelin-like mitochondria **(E,E”)** but barely an SSR membrane. White arrows indicate large clear endosomes, and white asterisk arrows show dark synaptic vesicles. Curves in panel D′ show detached T-bars with synaptic vesicles. **A’**,**B’**,**B”**,**C’**,**D’**, **E’** are enlargements of the white boxes in **A**,**B**,**B’**,**C**,**D**, **E**, respectively. **D”**, **E”** are enlargements of the black boxes in **D** and **E**, respectively. **C”** and **C”’** are enlargements of the big and small black boxes in **C**, respectively. Scale bars in **A**,**C’**,**C”**,**E**,**E”**: 200 nm; **A’**,**B**,**B”**,**D’**,**D”**:100 nm; **C**,**C”**,**D**: 500 nm.

*dnrx^83^* and *dnrx^174^* are hypomorphic mutants (Zeng et al., 2007) and live to adulthood, while *dnrx^273^* is a null mutant (Li et al., 2007), which is lethal during the pupal stage. Under electron microscopy, *dnrx*^273^ mutants had severe degeneration in NMJ boutons (Figures 4, 5), but the *dnrx^83/174^* mutant did not show a degeneration phenotype. In addition, SSR degeneration and obvious dark vesicles were not observed in axon terminals in *dnrx^83^*, *dnrx^174^* (data not shown), *dnrx^83/174^* (Figures 8A,A’), or *dnlg3* (Figures 7B,B’, 8B,B’’) mutants. However, NMJ bouton degeneration occurred in both the axon terminal and the SSR of the *dnrx^83^;dnlg3* double mutant (Figures 8C,E’). We observed dark vesicles in the axon terminal of *dnrx^83^;dnlg3* double mutants (Figures 8C,C’). The SSR retracted and formed a rare SSR membrane (Figures 8C,C’’), and a portion of the SSR was disordered (Figures 8C,C’’’). The sparse SSR membrane could form a large PSA outside of NMJs (Figure 8C). The T-bar structure was detached from the presynaptic membrane with clustered synaptic vesicles (Figures 8D,D’), and several dark lysosomes were observed in the PSA near the postsynaptic membrane (Figures 8D,D’’). A degenerating type Is bouton was observed that had almost no SSR membrane and contained poly-T-bars in a synapse, dark vesicles (Figures 8E,E’), and myelin-like autophagic vacuole (AV) that were severely damaged (Figures 8E,E’). The type Ib bouton had more large-sized clear vesicles in the *dnrx^83/174^* (Figures 8A,A’) and *dnlg3* (Figures 7B,B’, 8B,B’’) mutants, and the large clear endosomes further increased and collapsed inwardly in the *dnrx^83^;dnlg3* double mutant (Figures 8C,C,C’’’).

We statistically analyzed the above results based on electron microscopy data. The percentage of degenerated SSR and synaptic vesicle in wild-type NMJ boutons fluctuated significantly with different strains related to *dnrx* and *dnlg*s flies, and the detailed value of percentage was shown in Table 1. Since degenerated synaptic vesicles were tended to be regional, we also calculated the percentage of the area of degenerated synaptic vesicles in a single NMJ bouton, and the detailed value of percentage was shown in Table 1 and Figure 8F.

**Table 1:** Degenerated boutons (SSR and SV) analysis in TEM.

Genotype	*W^1118^*	*nrx^273^*	*nrx^273^RE*	*nrx^83^*	*nrx^83^; nrx^174^*	*nlg1*	*nlg2^KO70^*	*nlg2^KO70def^*	*nlg2^KO70^ RE*	*nlg3*	*nlg4*	*nrx^83^;nlg^3^*	*nlg2;nlg3*
Total Boutons	250	19	20	14	20	39	26	27	32	61	81	42	64
Boutons of DE SSR	7	9	3	4	3	6	5	2	1	1	15	6	4
Percentage of DE SSR Boutons	2.8	47.0	15.0	28.6	15.0	15.4	19.2	7.4	3.1	1.6	18.5	14.3	6.3
Boutons of DE SV	12	11	2	7	5	17	2	3	2	3	16	18	22
Percentage of DE SV Boutons	4.8	57.9	10.0	50.0	25.0	43.6	7.7	11.1	6.2	4.9	19.8	42.9	34.4
Percentage of DE SV Area	1.4 ± 0.5	27.6 ± 8.2	6.7 ± 5.2	22.8 ± 6.4	11.9 ± 4.8	48.7 ± 7.3	2.8 ± 2.0	8.7 ± 5.2	2.2 ± 1.6	4.1 ± 2.4	17.0 ± 4.0	26.6 ± 5.3	21.9 ± 3.9
Percentage of DE SV Area *p*-value	ns	****	ns	*(0.0162)	ns	****	ns	ns	ns	ns	****	****	****

^a^All fly strains and analyzes are described in section Materials and Methods, and all boutons are from the 6th/7th muscles. ^b^DE SSR: degenerated Subsynaptic Reticulum. ^c^DE SV: degenerated synaptic vesicles. ^d^The percentage of degenerated synaptic vesicles area. Using one-way ANOVA analysis. SEM, standard error of the mean. *p < 0.05; ****p < 0.0001. *p*-values for rate of degenerated synaptic vesicles area were determined by comparison to third instar wild-type(W^1118^).

### Synaptotagmin is not distributed in ultrafine particles in degenerated boutons

**Figure 9:**
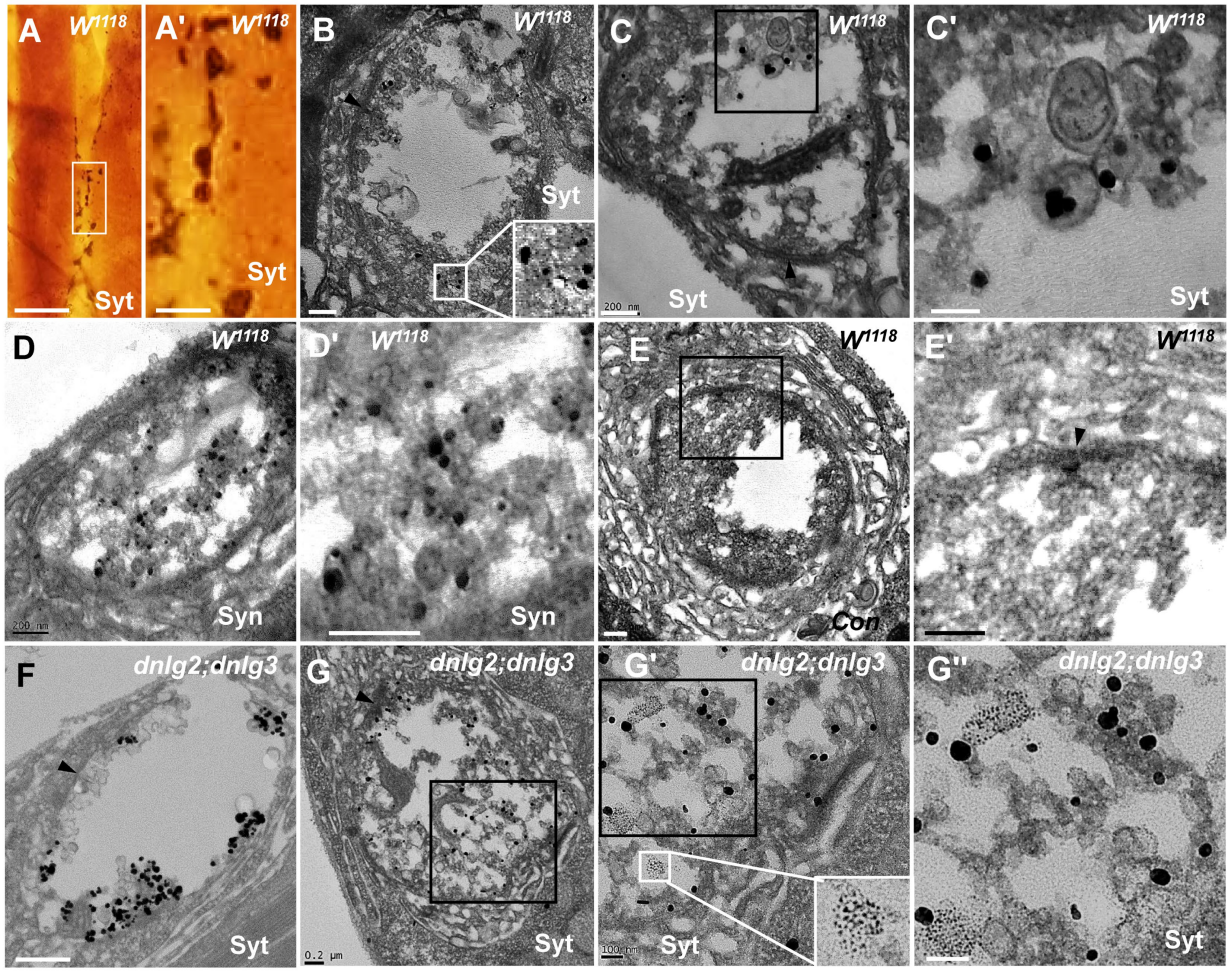
Synaptotagmin is not distributed in ultrafine particles in degenerated boutons Syt is present in the wild-type NMJ bouton under light microscopy **(A,A’)**, and Syt **(B,C’)** and Syn **(D,D’)** are present in the synaptic vesicles under TEM; (**E-E’**) is the control with preimmunoelectron microscopy. Syt is present in synaptic vesicles **(F)** but not in ultrafine particles of the degenerated NMJ bouton in *dnlg2;dnlg3* **(G,G”)**. Wedges show synapses or T-bars. **A’**,**C’**,**D’**,**E’**,**G’**, and **G”** are enlargements of the boxes in **A**,**C**,**D**,**E**,**G**, **G’**, respectively. Scale bars in **A**: 100 μm; **A’**: 20 μm; **B**,**C**,**D**,**E’**,**G**: 200 nm; **C’**,**G’**,**G”**: 100 nm; **F**: 500 nm.

Synaptotagmin (Syt) and synapsin (Syn) are synaptic vesicular proteins and have been used as markers of synaptic vesicles in *Drosophila* in many studies. Based on our above results, we propose the following scenario for synaptic vesicle degeneration: spherical clear synaptic vesicles collapse into dark vesicles and then fragment into 2–3 nm ultrafine particles. Syt was present in the NMJ bouton in wild-type flies under light microscopy (Figures 9A,A’), Syt (Figures 9B,C’) and Syn (Figures 9D,D’) were present in the synaptic vesicles in TEM, but Syt and Syn were not present in the control (Figures 9E,E’) evaluated with Pre-embedding immunogold electron microscopy.

Due to the obvious degeneration of NMJ boutons and the presence of ultrafine particles in the *dnlg2;dnlg3* double mutants, we investigated whether Syt was present in these ultrafine particles. Syt was present in synaptic vesicles of NMJ boutons without ultrafine particles (Figure 9F). However, Syt was not present in the ultrafine particles but was present in the synaptic vesicles in the degenerated NMJ boutons (Figures 9G,G”). Therefore, in the process of synaptic vesicle degeneration into ultrafine particles, synaptic vesicle-associated proteins, such as Syt, appeared to be completely degraded and could not be detected by the corresponding antibodies in TEM.

### Neurexin and neuroligins jointly regulate synapse degeneration at the neuromuscular junction

Syt was not observed via TEM in the ultrafine particles that were degeneration products, which indicates that the degenerated synaptic vesicles might lose the signaling of Syt and Syn proteins with the disintegration of synaptic vesicles. Next, we investigated whether the degenerated NMJ boutons could be observed via confocal microscopy with a 3D scanning function for biological samples. To facilitate the evaluation of degenerated NMJ boutons, we observed and counted type Ib boutons that had a larger size, and the synaptic vesicles were numerous and relatively dispersed in the outer layer of axon terminals with respect to the type Is boutons (Atwood et al., 1993; Jia et al., 1993).

**Figure 10:**
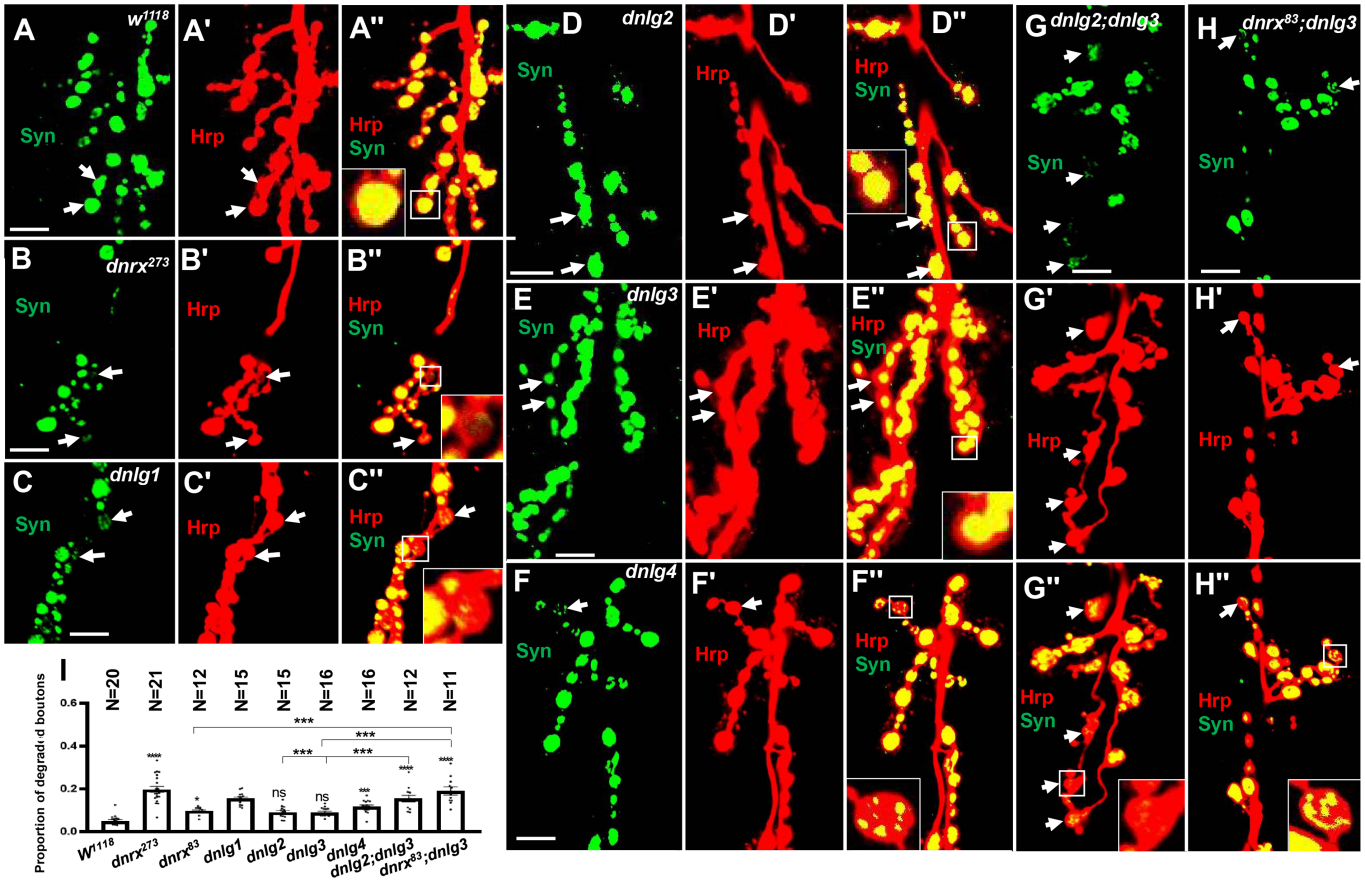
Almost no degeneration in type Ib bouton in wild-type **(A-A’’)** and *dnlg2* **(D-D’’)** and *dnlg3* **(E-E’’)**. The degenerated type Ib bouton in *dnrx^273^* **(B-B’’)**, *dnlg1* **(C-C’’)**, *dnlg4* **(F’’)**, *dnlg2;dnlg3* **(G-G’’)** and *dnrx83;dnlg3* **(F-F’’)**. Quantity statistics of degenerated type Ib bouton **(I)**. The white arrows show the degenerated type Ib bouton. Scale bars, 10 μm. **, P<0.01; ***, P<0.001; ns, no significance.

Most type Ib boutons had strong Syt (data not shown) and Syn (Figures 10A,A’’) protein signals at the 6th/7th muscles in the A_3_ or A_2_ segment in wild-type lines. The Syn signals in type Ib boutons were regular, globular and covered the entire axon terminal in large pinhole mode (we adjusted the pinhole to 600 to acquire thicker optical sections with a highly sensitive GaAsP detector and used an 80 pinhole for routine observation), and the proportion of degenerated type Ib boutons was very low (0.05 ± 0.01, *N* = 18). After the confocal microscopy focal length was adjusted, although speckled Syn signals were present at some optical sections, in the middle optical area, 2–3 sections were always filled with Syn signals in axon terminals (Figures 10A,A’’). There were very weak or no Syn signals in some type Is boutons under the same microscopy parameters, including pinhole size, laser intensity and image brightness.

However, several degenerated type Ib boutons could be observed in mutants (Table 2), and the proportion of degenerated type Ib boutons increased in *dnrx^273^*, (0.20 ± 0.01, *N* = 21; Figures 10B,B’’), *dnlg1* (0.15 ± 0.01, *N* = 15; Figures 10C,C’’), and *dnlg4* (0.12 ± 0.01, *N* = 16; Figures 10F,F’’). The criteria for judging the degenerated type Ib boutons were as follows: 1. the Syn signals were always very weak compared with those in other type Ib boutons (Figures 10B,B’’); 2. the Syn signals were always distributed in spots in the bouton (Figures 10C,C’’,F,F’’); and 3. after adjustment of the confocal microscope pinhole size, laser intensity and image brightness, the Syn signals in degenerate type Ib boutons faded (Figures 10B,B’’) or were distributed in small dots (Figures 10G,G’’) in most instances, but the Syn signals remained spherical and dense in other type Ib boutons.

**Table 2:** Degenerated boutons (SV) analysis in confocal.

Genotype	*W^1118^*	*nrx^273^*	*nrx^Δ83^*	*nlg1*	*nlg2^KO70^*	*nlg3^KO88^*	*nlg4^Δ10^*	*nlg2;nlg3*	*nrx^Δ83^;nlg3*
Boutons of DE SV (SEM)	2.61 ± 0.30	7.48 ± 0.46	4.42 ± 0.26	8.67 ± 0.46	4.67 ± 0.29	6.12 ± 0.34	6.56 ± 0.41	9.75 ± 1.16	11.36 ± 1.19
Boutons of DE SV *P*-value		****	ns	****	ns	***	****	****	****
Percentage of DE boutons (SEM)	0.05 ± 0.01	0.20 ± 0.01	0.10 ± 0.01	0.15 ± 0.01	0.10 ± 0.01	0.10 ± 0.004	0.12 ± 0.01	0.16 ± 0.02	0.19 ± 0.02
Percentage of DE boutons *P*-value		****	*	****	ns	ns	***	****	****

^a^All fly strains and analyzes are described in section Materials and Methods, and all boutons are from the 6th/7th muscles. ^b^DE SSR: degenerated Subsynaptic Reticulum. ^c^DE SV: degenerated synaptic vesicles. ^d^The percentage of degenerated boutons. Using one-way ANOVA analysis. SEM, standard error of the mean. *p < 0.05; ****p < 0.0001. P-values for rate of degenerated boutons were determined by comparison to third instar wild-type(W^1118^).

There were no obvious degenerate type Ib boutons in *dnlg2* and *dnlg3* (Table 2), the proportion of degenerated type Ib boutons was 0.09 ± 0.01, *N* = 15 (Figures 10D,D’’) in *dnlg2*, and 0.09 ± 0.005, *N* = 16 in *dnlg3* (Figures 10E,E’’), and the proportion of degenerated type Ib boutons increased in *dnrx^83^* which were partial mutants of whole *dnrx* genes. The in *dnrx^83^*, the proportion of degenerated type Ib boutons was 0.10 ± 0.01, *N* = 12 (Figures 10I,I’’). Interestingly, degenerate type Ib boutons were frequently found in *dnlg2;dnlg3* and *dnrx^83^;dnlg3* double mutants, and proportion of degenerated type Ib boutons significantly increased in *dnlg2;dnlg3* (0.16 ± 0.02, *N* = 12; Figures 10G,G’’) and *dnrx^83^;dnlg3* (0.19 ± 0.05, *N* = 11; Figures 10I,I’’). Therefore, the synaptic vesicle-associated protein Syn could be detected with the corresponding antibodies via confocal microscopy, and the degeneration of terminals was accelerated in *dnrx* and *dnlgs* mutants, which was highly consistent with the results obtained under electron microscopy (Table 1; Figure 8F).

## Discussions

### Ultrastructural features of NMJ bouton degeneration in third-instar *Drosophila* larvae

Synaptic degeneration can be caused by degenerative neurological diseases, such as Alzheimer’s disease (Jaworski et al., 2011; Dominguez-Alvaro et al., 2018) and prion disease (Siskova et al., 2009; Caleo et al., 2012); it can also be caused by injuries, such as surgery, microwaves (Tan et al., 2017) and mild fluid percussion (Powell et al., 2018) and can occur in aging animals (Gillingwater et al., 2002; Fan et al., 2018). Fragmentation and degeneration of organelles exposes more proteins or polypeptides, which contain amino groups to which osmic acid can easily adsorb, resulting in electron density under an electron microscope. According to the current literature (Jaworski et al., 2011; Qi et al., 2017; Li et al., 2018), degeneration of neuronal cytoplasm and synaptic boutons is characterized by dark electron density under an electron microscope.

The *Drosophila* larval NMJ is a powerful experimental model, and it contains three bouton types: type I, type II and type III. Type I boutons are often used for studying synaptic development, signal transmission and neurological disease and are repeatedly wrapped by the SSR that is formed by the muscle cell membrane, and the synapse, T-bar structures, synaptic vesicles, mitochondria, and cytoskeleton appear in the axon terminal. Type I boutons include type Ib (big) and type Is (small). At present, ultrastructural phenotype analysis of type I boutons has mainly focused on T-bars, synaptic vesicles and the SSR (Xing et al., 2014; Zhang et al., 2017), and few studies (Gan and Zhang, 2018) have analyzed ghost boutons, which are indicators of poor bouton development, hypogenetic boutons, or satellite boutons and thus depict synaptic overgrowth. Electron microscopy is a powerful tool to study synapse structure (Glausier et al., 2019). In the present study, we describe the ultrastructural characteristics of *Drosophila* larval NMJ bouton degeneration, primarily based on the dark electron density observed via electron microscopy.

The degeneration of *Drosophila* NMJ boutons included collapse and fragmentation of synaptic vesicles, retraction and degradation of the SSR, and deformation of the profile. The normal synaptic vesicles were globular, clear, and small (approximately 35 nm diameter), with a single membrane layer. The clear synaptic vesicles collapsed into dark synaptic vesicles and then fragmented into ultrafine particles during the process of terminal degeneration. We deduced that this process involves the following steps: globular, clear synaptic vesicles collapse out or form an irregular, larger or smaller profile with membrane laceration. This allows lipids and proteins in the vesicles to be fully exposed and easily stained by heavy metals, such as osmium, acetic acid and lead citrate; thus, the dark synaptic vesicles are electron dense under an electron microscope. Then, the dark synaptic vesicles are further degraded and fragmented into ultrafine particles. Neurofilament bundles accumulate in the degenerating neuronal cytoplasm (Gillingwater et al., 2002; Siskova et al., 2009), similar to lysosome accumulation (Siskova et al., 2009) and neurofilament degeneration in injured brains (Qi et al., 2017). We observed intrusion of disturbed microtubules into NMJ boutons where actin and synaptic vesicles should be located, and dark synaptic vesicles formed at the end of microtubules in the boutons. In *dnlg1* mutants, dark synaptic vesicles also occurred along the long axon. Therefore, the fragmentation of synaptic vesicles was associated with abnormal assembly and transport of microtubules. Furthermore, the NMJ boutons contained autophagosomes (Figures 6H,H’; Li et al., 2018) and abnormal mitochondria (Figures 8E,E’’), which are associated with synaptic degeneration (Siskova et al., 2009). NMJ boutons also contained presynaptic degradation products, including presynaptic organelle components, cytoskeleton components and T-bar components, and degenerated together and gathered in dark clumps (Figures 4A,A’’) in the axon terminal; we were unable to completely distinguish the morphological structure of organelles (Caleo et al., 2012).

Presynaptic organelles appear swollen or dark and dense (Qi et al., 2017), and degenerating dendrites are also dark (Jaworski et al., 2011) during synapse degeneration. Once the synapse is degenerated, the PSD looks curved (Caleo et al., 2012), and both presynaptic and postsynaptic membranes have been shown to become thin in aging mice (Fan et al., 2018; Figures 1G,G”). During NMJ bouton degeneration in wild-type *Drosophila*, the complex SSR membrane became swollen and retracted until a few remnants were left, but more SSR remnants degenerated into fragments or thin slices in *dnrx* mutants (Figures 4, 5). Degeneration of NMJ boutons starts from the SSR membrane, but the rate of SSR membrane degeneration is slower than that of the axon terminal. Degeneration of SSR membranes primarily manifested as retraction, and synaptic vesicles and presynaptic organelles primarily showed lysis and collapse.

With degeneration of the presynaptic terminal and postsynaptic SSRs, NMJ boutons lost their globular spherical profile and become irregular, with a small profile.

The degenerated synaptic boutons are engulfed by Schwann cells (Gillingwater et al., 2002; Smith et al., 2013; Lee et al., 2016; Jung et al., 2019) in mammalian NMJs, but degenerated NMJ boutons are not eliminated by glial or muscle cells in *Drosophila*. Therefore, degenerated NMJ boutons degenerate *in situ* and appear to be abandoned in the muscles because most organs, including muscles and the NMJ system, are completely autolyzed in the next pupal stage.

### Neuromuscular junction bouton development or degeneration: ultrastructural differences

The number of NMJ boutons increases 10-fold from the first instar to the third instar in *Drosophila* (Schuster et al., 1996), and degenerated NMJ boutons can be seen as remnants of the pruned NMJ boutons during NMJ bouton development. The degenerated NMJ boutons were rare in wild-type flies, which meant that most pruned NMJ boutons produced physiological retraction, firming the synaptic footprint (Eaton et al., 2002; Eaton and Davis, 2005).

Axon terminals were small, synaptic vesicles were dark, and postsynaptic SSRs were loose and thin in degenerating NMJ boutons, which happens to be a feature of developing NMJ boutons. Thus, degenerating and developing NMJ boutons must be distinguished from each other. In degenerating NMJ boutons, the speckled membrane of clear vesicles, dark vesicles and dark clumps without a membrane, dark ultrafine particles, synaptic vesicles and mitochondria were reduced; the T-bar was detached from the presynaptic membrane; and mitochondria were swollen, with few and fractured cristae (Qi et al., 2017; Fan et al., 2018). Furthermore, the SSR was reduced and irregularly disorganized, with a large PSA. In developing neurite, the dark synaptic vesicles are spherical and wrapped in a complete biofilm (Gan et al., 2014).

### DNrx and DNlgs cause NMJ bouton degeneration

The synaptic adhesion molecule Nrx is mainly located in the presynaptic membrane, and Nlg is mainly distributed in the postsynaptic membrane. Nrx and Nlg defects lead to autism and neurological disorders. There is 1 *dnrx* gene and 4 *dnlg* genes in *Drosophila*, and the current studies on *dnrx* (Li et al., 2007; Rui et al., 2017) and *dnlg1-4* (Banovic et al., 2010) have focused on synaptic signaling and synapse development (Gan and Zhang, 2018) in NMJ boutons.

Here, our results showed that *dnrx* and *dnlgs* caused NMJ bouton degeneration in *Drosophila*. Neurodegenerative diseases are accompanied by severe synaptic degeneration (Jaworski et al., 2011; Qi et al., 2017; Dominguez-Alvaro et al., 2018). The NMJ boutons showed severe degeneration in the *dnrx^273^*, *dnlg1*, and *dnlg4* mutants, but there was no obvious synaptic degeneration in the *dnrx^83^*, *dnlg2* or *dnlg3* mutants. However, there was obvious synaptic degeneration in the *dnlg2;dnlg3* and *dnrx^83^/dnlg2* double mutants, which further confirms synergistic functions between *dnrx* and *dnlgs* and between *dnlg2* and *dnlg3*.

*dnrx* and *dnlgs* mutations interfere with the BMP (bonemorphogenetic protein) and Wnt (wingless-int) signaling pathways by disrupting spectin (Xing et al., 2018), actin (Rui et al., 2017) and microtubules (Banerjee et al., 2017), both of which are essential cytoskeletal components necessary for synapse formation and development. Mutations in members of the BMP pathway, such as *wit* and *gbb* (Aberle et al., 2002; Marques et al., 2002), have been shown to cause abnormal presynaptic ruffles that are similar to those observed in *dnrx* and *dnlgs* mutants (Banovic et al., 2010; Banerjee et al., 2017; Zhang et al., 2017) and to cause presynaptic shedding of T-bars (Aberle et al., 2002), similar to that observed in *dnrx* ^273^ mutants (Figure 5) via TEM. The *dnlg4* gene and other gene members of the BMP pathway, such as *wit*, *tkv*, and *mad*, have been found to have a dose-dependent genetic interaction in NMJ development (Zhang et al., 2017).

Wnt signaling is involved in the regulation of synaptic morphology and functional plasticity, and Wnt deficiency is closely related to Alzheimer’s disease (Tapia-Rojas and Inestrosa, 2018). Blocking the secretion of Wingless (Wg, a Wnt homolog in flies) at a certain stage of development inhibits the growth of boutons by destroying the presynaptic microtubule skeleton (Packard et al., 2002). We also found abnormal microtubule skeletons in *dnlg1* mutants (Figure 6C), which could transport other organelles, and microtubules in *dnrx* mutants were shown to be broken (Banerjee et al., 2017). Recent studies have shown that Nrx and Nlg defects cause or worsen neurodegenerative diseases, and the expression levels of Nlg and Nrx are significantly downregulated in Alzheimer’s disease (Martinez-Mir et al., 2013; Sindi et al., 2014), which suggests that Nrx and Nlg are associated with the synaptic degeneration that occurs in *Drosophila* NMJ boutons.

Therefore, in studying the autism caused by *nrxs* and *nlgs,* attention should be given to not only research on classic synaptic signal transmission and on the developmental balance of synaptic boutons (Gan and Zhang, 2018) but also synaptic degeneration, which could also cause abnormal synaptic signal transmission.

It was worth noting that the degenerated NMJ boutons originated from swelling and retraction of the SSR membrane in wild type flies (Figure 3), but the significant degenerated synaptic vesicles were in NMJ boutons along with the relatively intact SSR in *dnlg1* mutants (Figures 6A,A’C,C’), which predicted that the presynaptic degeneration (synaptic vesicles) and the postsynaptic degeneration (SSR) might have different regulatory mechanisms.

### Neuromuscular junction bouton degeneration patterns in *Drosophila*

**Figure 11:**
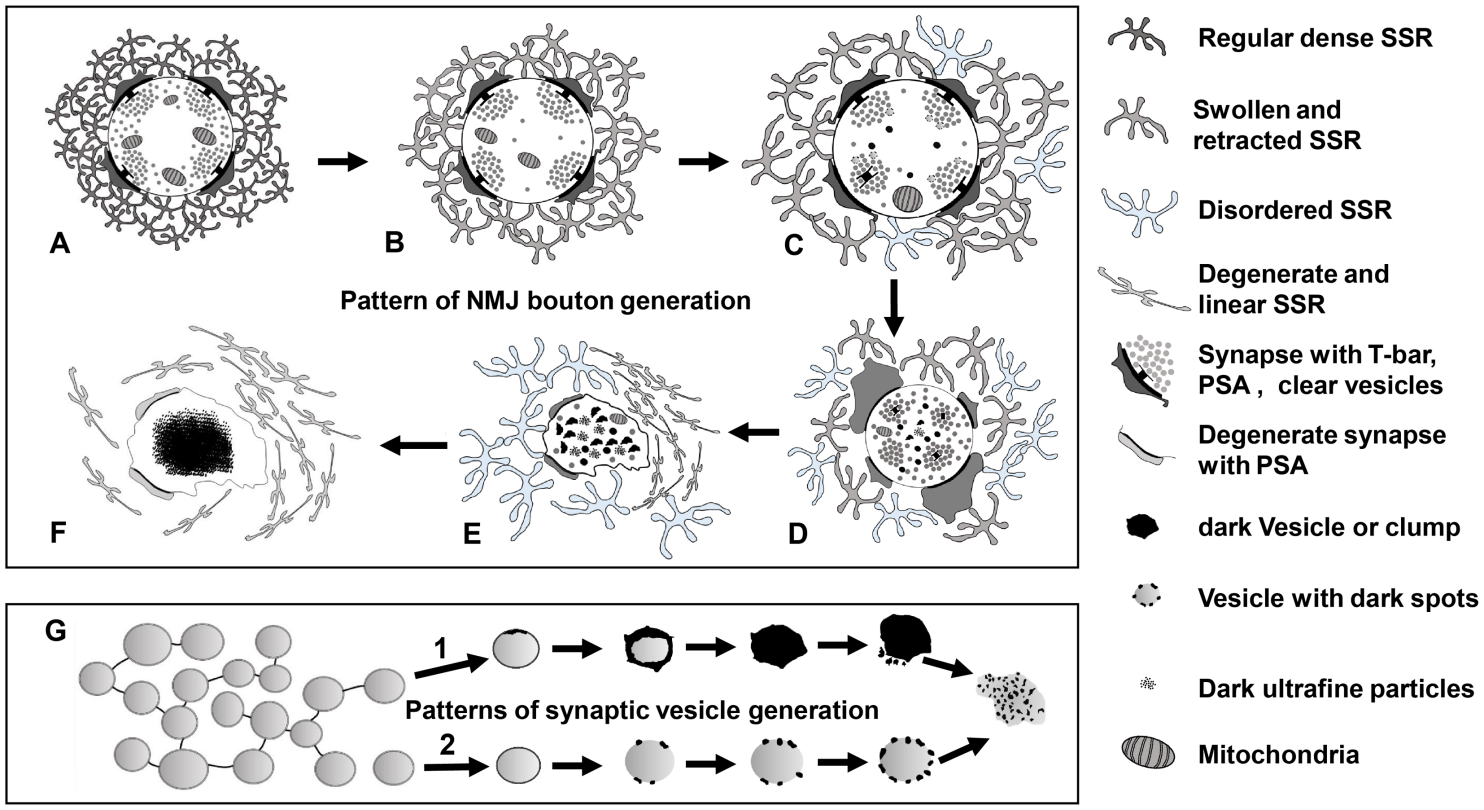
The degeneration model for *Drosophila* NMJ synapses A type Ib bouton contains T-bars, dense clear synaptic vesicles, mitochondria, and a regular SSR with PSA **(A)**. The SSR membranes start to swell and retract, and mitochondria and synaptic vesicles are reduced **(B)**. The T-bar detaches with the swelling mitochondria, degenerating vesicles appear in the center of the terminal, and SSR undergoes further swelling and retraction **(C)**. All the T-bars detach with clusters of clear synaptic vesicles, more dark vesicles deform and collapse into irregular dark vesicles without membranes and then fragment into dark ultrafine particles, and the SSR is more retracted and fluffy with PSA expansion or reduction **(D)**. The terminal deforms with more collapsed dark vesicles and residual synaptic vesicles, mitochondria and PSA, and most of the SSR become loose and disordered, some of which degenerates into a linear morphology **(E)**. All residual membranes become very thin or degenerate into a linear form, and all the residual elements in the terminal degenerate *in situ* and eventually form a cluster of ultrafine particles **(F)**. There are two modes of synaptic vesicle degeneration **(G)**. First, the synaptic vesicles without actin filaments degenerated on the membrane with ultrafine spots and collapsed and dispersed, exhibiting an irregular profile and dark ultrafine particles. Second, the clear synaptic vesicle with actin filament degenerated into a dense synaptic vesicle, formed irregular dark clumps without a membrane, and collapsed and dispersed, exhibiting an irregular profile and dark ultrafine particles. NMJ bouton degeneration occurs in normal physiological conditions, but it is accelerated in *dnrx* and *dnlgs*. Furthermore, a synergistic effect exists in *dnrx*;*dnlgs* and *dnlg2*;*dnlg3* double mutants to promote NMJ bouton degeneration.

According to our findings and the current literature, we propose a model of NMJ bouton degeneration (Figure 11). Type Ib boutons contain T-bars, dense clear synaptic vesicles, mitochondria, and a regular SSR with a narrow PSA (Figure 11A). After NMJ bouton degeneration, SSR membranes begin to swell and retract, and some organelles, such as mitochondria and synaptic vesicles, are transported away from the terminal and reduced (Figure 11B; Fan et al., 2018). This phenomenon is accompanied by reduction and fading of the presynaptic cytoskeleton-associated molecule Futsch (Keller et al., 2011; Xiong and Collins, 2012) from distal boutons, which causes presynaptic contraction of the synapse and forms a synaptic footprint that has a smaller axon terminal and fewer synaptic vesicle signals (Eaton et al., 2002; Eaton and Davis, 2005). Then, the T-bar detaches from the presynaptic membrane (Aberle et al., 2002) with the swelling of mitochondria (Tan et al., 2017), some degenerating dark vesicles appear in the center of terminals, and the SSR further swells and retracts (Figure 11C). All the T-bars detach from the presynaptic membrane and cluster the clear synaptic vesicles, more dark vesicles deform and collapse into irregular dark vesicles without a biofilm and then fragment into dark ultrafine particles (Figures 1, 2), and the SSR becomes more retracted and fluffy, along with PSA expansion or reduction (Figure 11D). Then, the terminal deforms, with more collapsed dark vesicles and residual synaptic vesicles, mitochondria and PSA, and most of the SSR becomes loose and disordered, some of which degenerates into a linear morphology (Figure 11E). Last, all residual membranes, including presynaptic and postsynaptic membranes (Fan et al., 2018), become very thin; most of the SSR degenerates into a linear shape; and all the residual elements in axon terminals, such as synaptic vesicles, mitochondria, cytoskeleton components, and T-bars, degenerate *in situ* and eventually form a cluster of ultrafine particles (Figure 11F).

Axon terminals and elements within axon terminals degenerate with the postsynaptic SSR. However, swelling and retraction of the SSR occurs prior to axon terminal degeneration, which is faster and more intense than SSR degeneration. Furthermore, the degeneration of synaptic vesicles begins at the center of terminals and follows two specific degeneration procedures (Figure 11G).

NMJ bouton degeneration occurs under normal physiological conditions but is accelerated in *dnrx* and *dnlgs* mutants. Furthermore, there is a synergistic effect in *dnrx*;*dnlgs* and *dnlg2;dnlg3* double mutants that promotes NMJ bouton degeneration (Figures 7, 8, 10).

## Conclusion

NMJ bouton degeneration occurs under normal physiological conditions but is accelerated in *dnrx* and *dnlgs* mutants. Furthermore, a synergistic effect exists between *dnrx*;*dnlgs* and *dnlg2;dnlg3* double mutants that promotes degeneration of NMJ boutons, suggesting that both neurexins and neuroligins play a vital role in preventing synaptic degeneration. This study proposes a model of NMJ bouton degeneration patterns, which is very conducive to the in-depth study of neurodegeneration.

## Data availability statement

The original contributions presented in the study are included in the article/supplementary material, further inquiries can be directed to the corresponding author.

## Ethics statement

The research involved animals, which were all bred in the animal facility at Southeast University. All experiments were performed according to guidelines approved by Southeast University, and no informed consent was required for this research.

## Author contributions

GG: conceptualization, Funding acquisition, Writing – original draft, Investigation. CM: Data curation, Formal analysis, Writing – review & editing. YQ: Data curation, Funding acquisition, Writing – review & editing. GX: Funding acquisition, Investigation, Writing – review & editing. ZC: Methodology, Writing – review & editing. SQ: Conceptualization, Writing – review & editing, Data curation, Formal Analysis. XW: Writing – review & editing, Conceptualization. GJ: Data curation, Formal analysis, Investigation, Writing – review & editing.

## Abbreviations

NMJ: neuromuscular junction
SSR: subsynaptic reticulum
PSA: postsynaptic area
*dnrx*: *dneurexin*, *Drosophila neurexin*,
*dngl*: *dneuroligin*, *Drosophila neuroligin*
TEM: transmission electron microscopy
BMP: bone morphogenetic protein
Wnt: wingless-int
